# MicroRNA-146a suppresses tumor malignancy via targeting vimentin in esophageal squamous cell carcinoma cells with lower fibronectin membrane assembly

**DOI:** 10.1186/s12929-020-00693-4

**Published:** 2020-11-28

**Authors:** Hong-Yi Chang, Chi-Hua Lee, Yi-Syuan Li, Jing-Tong Huang, Sheng-Hui Lan, Yi-Fang Wang, Wu-Wei Lai, Yi-Ching Wang, Yan-Ju Lin, Hsiao-Sheng Liu, Hung-Chi Cheng

**Affiliations:** 1 Department of Biotechnology and Food Technology, College of Engineering, Southern Taiwan University of Science and Technology, Tainan, Taiwan; 2 Department of Microbiology and Immunology, College of Medicine, National Cheng Kung University, Tainan, Taiwan; 3 Institute of Basic Medical Sciences, College of Medicine, National Cheng Kung University, Tainan, Taiwan; 4 Department of Life Sciences and Institute of Genome Sciences, National Yang-Ming University, Taipei, Taiwan; 5 Department of Biochemistry and Molecular Biology, College of Medicine, National Cheng Kung University, Tainan, Taiwan; 6 Division of Thoracic Surgery, Department of Surgery, National Cheng Kung University Hospital, College of Medicine, National Cheng Kung University, Tainan, Taiwan; 7 Department of Pharmacology, College of Medicine, National Cheng Kung University, Tainan, Taiwan; 8 Biomedical Technology and Device Research Laboratories, Industrial Technology Research Institute, Hsinchu, Taiwan; 9 M. Sc. Program in Tropical Medicine, College of Medicine, Kaohsiung Medical University, Kaohsiung, Taiwan; 10 Center for Cancer Research, Graduate Institute of Clinical Medicine, College of Medicine, Kaohsiung Medical University, Kaohsiung, Taiwan

**Keywords:** ESCC, *miR-146a*, Vimentin, Cell migration, Invasion

## Abstract

**Background:**

Esophageal squamous cell carcinoma (ESCC) is widely prevalent in Taiwan, and high metastatic spread of ESCC leads to poor survival rate. Fibronectin (FN) assembly on the cell membrane may induce ESCC mobility. MicroRNAs (MiRNAs) are abundant in and participate in tumorigenesis in many cancers. However, the role of MiRNA in FN assembly-related ESCC mobility remains unexplored.

**Methods:**

We divided ESCC CE81T cells into high-FN assembly (CE81^FN+^) and low-FN assembly (CE81^FN−^) groups by flow cytometry. MiRNA microarray analysis identified *miR-146a* expression as the most down-regulated miRNA in comparison of CE81^FN+^ and CE81^FN−^ cells.

**Results:**

Cell proliferation and migration were decreased when CE81^FN+^ cells overexpressed transgenic *miR-146a* compared to the parental cells, indicating an inverse correlation between low *miR-146a* expression and high proliferation as well as motility of FN assembly ESCC cells. Furthermore, vimentin is the target gene of *miR-146a* involved in ESCC tumorigenesis. *MiR-146a* suppressed cell proliferation, migration and invasion of CE81^FN+^ cells through the inhibition of vimentin expression, as confirmed by real-time PCR, Western blotting and Transwell™ assay. Analysis of one hundred and thirty-six paired ESCC patient specimens revealed that low *miR-146a* and high vimentin levels were frequently detected in tumor, and that the former was associated with late tumor stages (III and IV). Notably, either low *miR-146a* expression or high vimentin level was significantly associated with poor overall survival rate among ESCC patients.

**Conclusions:**

This is the first report to link FN assembly in the cell membrane with *miR-146a*, vimentin and ESCC tumorigenesis both in vitro and in ESCC patients.

## Background

Esophageal cancer is the fifth most common cancer in males and the ninth leading cause of cancer death in Taiwan [1]. Two major histological types of esophageal cancers are esophageal squamous cell carcinoma (ESCC) and adenocarcinoma (EA). Over 95% of predominant esophageal cancers in Taiwan are classified as ESCC and its incidence continues to increase [2]. In addition, the prognosis of ESCC is poor and 5-year survival rate is less than 15%, due to the high rates of tumor invasion and metastasis [3–5]. The progression of metastasis in ESCC is complex, involving extracellular matrix (ECM)-cell interaction, cell signaling network, gene regulation and epithelial mesenchymal transition (EMT). These processes turn benign tumor cells into malignant ones. However, the mechanism whereby the regulation of ECM-affected EMT beings about deterioration of cell behaviors during carcinogenesis is complex and poorly understood.

Fibronectin (FN) is an adhesive glycoprotein protein present in ECM and plasma, which also functions as a mesenchymal marker involved in EMT-related signaling pathways [6–8]. Overexpression of FN has been reported in the malignant development of various cancer types including breast, lung, melanoma, colorectal, and ovarian cancers [7, 9–11]. Up-regulation of FN is also associated with ESCC tumor progression and the degree of ESCC tumor invasion [12]. Emerging evidence indicates that early cancer metastasis is activated by the interaction of ECM with the tumor cell surface receptors, such as FN-integrin interaction [13]. Overexpression of FN together with vimentin is associated with advanced stage and poor prognosis of ESCC patients [14]. Vimentin and FN are the markers of epithelial to mesenchymal transition (EMT), which participate in tumor cell migration and invasion [6]. Recent study indicated that EMT may be regulated by miRNAs, such as *miR-1199-5p* [15]. However, the relationship among FN assembly, miRNA and target genes during ESCC tumorigenesis remains unclear. This study aimed to clarify the role of microRNAs and target genes in ESCC tumorigenesis under low and high FN assembly conditions.

MiRNAs are small, noncoding, single-stranded RNA molecules harboring 20–23 nucleotides, which post-transcriptionally regulate the target gene expression in diverse physiological or pathological processes through the degradation of mRNAs or blockage of translation by annealing to the complementary mRNA coding sequences [6, 16, 17]. Dysfunction of miRNA regulation affects cellular homeostasis and triggers various diseases including cancers. MiRNA may function either as an oncogene or a tumor suppressor depending on its target gene [12, 18]. Therefore, whether miRNAs participate in FN-related tumorigenesis warrants further exploration. *MiR-146a* functions either as a tumor suppressor or an oncogene depending on the types of cancer cells [19]. There has been reported that *miR-146a* as a tumor suppresser is significantly decreased in both cancerous tissue and serum of ESCC [20]. However, the role of *miR-146a* and its target genes have not been characterized in ESCC.

Here, we showed that polymeric fibronectin assembly on the cell membrane promotes cell motility through the regulation of *miR-146a* and the target gene vimentin. Vimentin functions as a mesenchymal marker and participates in EMT. We confirmed that overexpression of *miR-146a* significantly suppresses cell proliferation, colony and tumor formation, as well as migration and invasion through inhibition of vimentin. Finally, the results of our analysis of clinical ESCC specimens support the notion that suppression of *miR146a* and up-regulation of vimentin promotes ESCC tumorigenesis.

## Methods

### Stable cell lines, cell culture and construction of vimentin 3′UTR luciferase reporter plasmid

CE81^FN+^ and CE81^FN−^ cells were sorted from a human ESCC cell line CE81T (ATCC® HTB­56™) by flow cytometry. CE81T cells incubated with anti-FN antibody-conjugated magnetic beads and Magnetic bead-bound CE81T cells were designated as CE81^FN+^ cells. The FN unbound cells were named as CE81^FN−^ cells. For the quantification purpose, the sorted cells stained with anti-FN polyclonal antibodies and analyzed by flow cytometry. CE81^FN+^ + *miR-146a* and CE81^FN+^ + CON stable cell lines were established using lentiviral infection from the parental CE81^FN+^ cells. The above cell lines and human embryonic kidney 293T cells were maintained in Dulbecco’s modified Eagle’s medium (DMEM; Gibco, Maryland, USA) containing 10% fetal bovine serum (FBS; Biological Industries, Kibbutz Beit haemek, Israel), penicillin (200 U/ml; Sigma, Missouri, USA) and streptomycin (100 μg/ml; Sigma) at 37 °C in a 5% CO_2_ incubator. ESCC cell lines KYSE150 (RRID: CVCL_1348) and KYSE70 (RRID: CVCL_1356) were cultured in RPMI1640 medium (Gibco). For construction of vimentin 3′-UTR luciferase reporter plasmid, the pMIR-REPORT™ (Thermo Fisher Scientific, Illinois, USA) was used following the manufacturer’s instructions. The target sequences of 3′-UTR region of wild- and mutant-type vimentin are provided in the Additional file 1: Table S1.

### Transfection

Stable CE81^FN+^ cell lines overexpressing *miR-146a* or miR-control were established by transfection with pre-*miR-146a* (Pre-miR™ miRNA precursor; Applied Biosystems, Massachusetts, USA) or pre-miR-control (5 μmole/L) using lentiviral infection. Transient transfection of anti-*miR-146a* (100 pmol/L) (Anti-miR™ miRNA inhibitor; Applied Biosystems), siRNA-vimentin (Invitrogen, California, USA), plasmid pcDNA3-vimentin (a gift from Dr. Ming-Der Perng, National Tsing-Hua University, Hsinchu, Taiwan) were transfected by Lipofectamine 2000™ following the manufacturer’s instructions (Invitrogen). The negative small interfering RNA controls used were pre-miRNA (Applied Biosystems), anti-miRNA (Applied Biosystems), and si-RNA (Invitrogen). The plasmid pcDNA3-Luc was used as a vector control. The cells (5 × 10^5^/well) were transfected with the above materials in a six-well plate for 48 h.

### Western blotting

Cells were lysed and protein samples were collected by centrifugation at 13,600 rpm for 20 min at 4 °C. The concentration of protein samples was determined by the Coomassive protein assay kit (Thermo Fisher Scientific). An equal amount of protein was loaded and separated by sodium dodecyl sulfate-polyacrylamide gel electrophoresis (SDS-PAGE). The separated protein bands were electrically transferred to a PVDF membrane (MILLIPORE, Massachusetts, USA). The membrane was blocked with 5% non-fat milk in Tris-buffered saline and Tween 20 (TBST) at RT for 1 h and then incubated with the specific primary antibodies. The following antibodies were used: vimentin (1:1000, MILLIPORE) and β-actin (1:5000, Sigma). The blots were incubated with ECL (Millipore) and captured by BioSpectrum AC (UVP). The results of Western blotting were quantified by density analysis using Vision Works TM LS image acquisition and analysis software.

### miRNA detection and real-time polymerase chain reaction (PCR)

Total RNA was extracted from tissues or cultured cells using the Trizol™ reagent (Invitrogen) according to manufacturer's instructions. The RNA pellet dissolved in DEPC water, and Ncode™ VILO™ cDNA synthesis miRNA kit (Invitrogen) was used for cDNA synthesis. The cDNA synthesized using MultiScribe™ Reverse Transcriptase in the presence of a universal Reverse primer (Invitrogen). The conditions of the thermal cycler were programmed as follows: 25 °C 10 min, 37 °C 120 min, 85 °C 5 min and finally at 4 °C. Real-time PCR was conducted to amplify the cDNA with SYBR Green SuperMix™ reagent (Invitrogen), the universal primer and *miR-146a*-specific primer (Additional file 1: Table S1). The mixture (20 μl) was loaded into a 96-well plate and analyzed by using a real-time PCR machine (Applied Biosystems). Data were normalized with endogenous U54, and the relative expression was calculated with the formula 2 − ΔCt values. For identifying the potential target gene of *miR-146a,* two groups were designed for this experiment. First group CE81^FN+^ cells transfected with N.C. and mimic-*miR-146a* and another group is CE81^FN+^ + CON and CE81^FN+^ + 146a stable cell lines. The comparison of these two gene expression profiles by Illumina™ oligonucleotide microarray analysis. The most up and down-regulated expression profile was represented in Table 2. Furthermore, only six potential targeting genes (VIM, IGSF1, FBXL10, CASK, PBX2, and UHRF1) were found after using three software programs, including Targetscan, Microcosm, and miRNAMap. Detailed information of the primer sequences for real-time PCR is listed in Additional file 1: Table S1.

### Transwell™ migration and invasion assay

Cell migration and invasion were investigated using Transwells with pore size of 8 μm (Falcon, BD Labware, Massachusetts, USA). For cell migration, the cells (10^6^ cells/ plate) were seeded into the upper chamber with 200 μl of serum free DMEM, and 600 μl of DMEM supplemented with 10% FBS was seeded into the lower chamber. For cell invasion, the upper chamber was coated with a 1 mg/ml matrigel (BD Biosciences, California, USA) in advance and incubated at 37 °C for 1 h. The cells were seeded into the upper chamber and incubated at 37 °C in a 5% CO_2_ incubator for 48 and 108 h, respectively. After incubation, the upper chamber was carefully removed using cotton swabs, and the cells that migrated to the bottom chamber were washed twice, fixed with 1% formaldehyde for 15 min and stained with 0.1% crystal violet (Sigma) for 10 min at RT. The migrated cells were counted in 10 randomly selected fields under a light microscope.

### Immunohistochemistry (IHC) and immunofluorescence staining (IFA)

CE81^FN+^, CE81^FN−^, CE81^FN+^ + CON and CE81^FN+^ + 146a cells were seeded (4 × 10^4^ cells/well) onto glass coverslips in a 6-well plate. The cells were fixed in 3.7% paraformaldehyde for 30 min, and washed by 1X PBS for 5 min followed by treatment with 0.1% Triton-X-100 for 30 min. Cells were blocked in 1X blocking buffer for 1 h at RT, and then treated with the monoclonal anti-vimentin antibody (Abcam, Cambridge, UK) for 16 h at 4 °C. After PBS washing for 10 min, cells were treated with secondary mouse monoclonal antibody and kept in the dark for 1 h. After PBS washing, cells were counterstained with Hoechst 33342 staining solution (Abcam) for 30 min and then mounted with glycerol gelatin (Sigma). The protein level of vimentin was evaluated by IHC staining of tumor and non-tumor tissues of the ESCC patient specimens. The anti-vimentin monoclonal antibody (Abcam) was used to detect vimentin protein. The secondary antibody was used followed by Streptavidin labeling (Dako, Cytomation, Carpinteria, USA). The slides were then treated with AEC solution for 20 min at RT, and counterstained with 10% hematoxylin (Muto Pure Chemicals, Tokyo, Japan) and mounted with glycerol gelatin (Sigma).

### ESCC patient specimens

One hundred and thirty six ESCC specimens were analyzed in this study. Informed consent was obtained from all patients and the study was approved by the Institutional Review Board, National Cheng Kung University Hospital, Tainan, Taiwan. All clinical samples were used in accordance with the guidelines of the Declaration of Helsinki. A total of 68-paired ESCC specimens were used for the detection of miRNA expression by real-time PCR, and a different set of 68 paired ESCC specimens were used for staining of *miR-146a* expression by in situ hybridization and vimentin expression by IHC and ISH staining (Table 3).

### Statistical analysis

Two-tailed Student’s t test was used. Data are shown as mean ± SD. Overall survival curves were calculated according to the Kaplan–Meier method (log-rank test). *p* < 0.05 was considered to be statistically significant.

## Results

### FN assembly on ESCC cell membrane correlated with *miR-146a* expression and cell migration

**Fig. 1:**
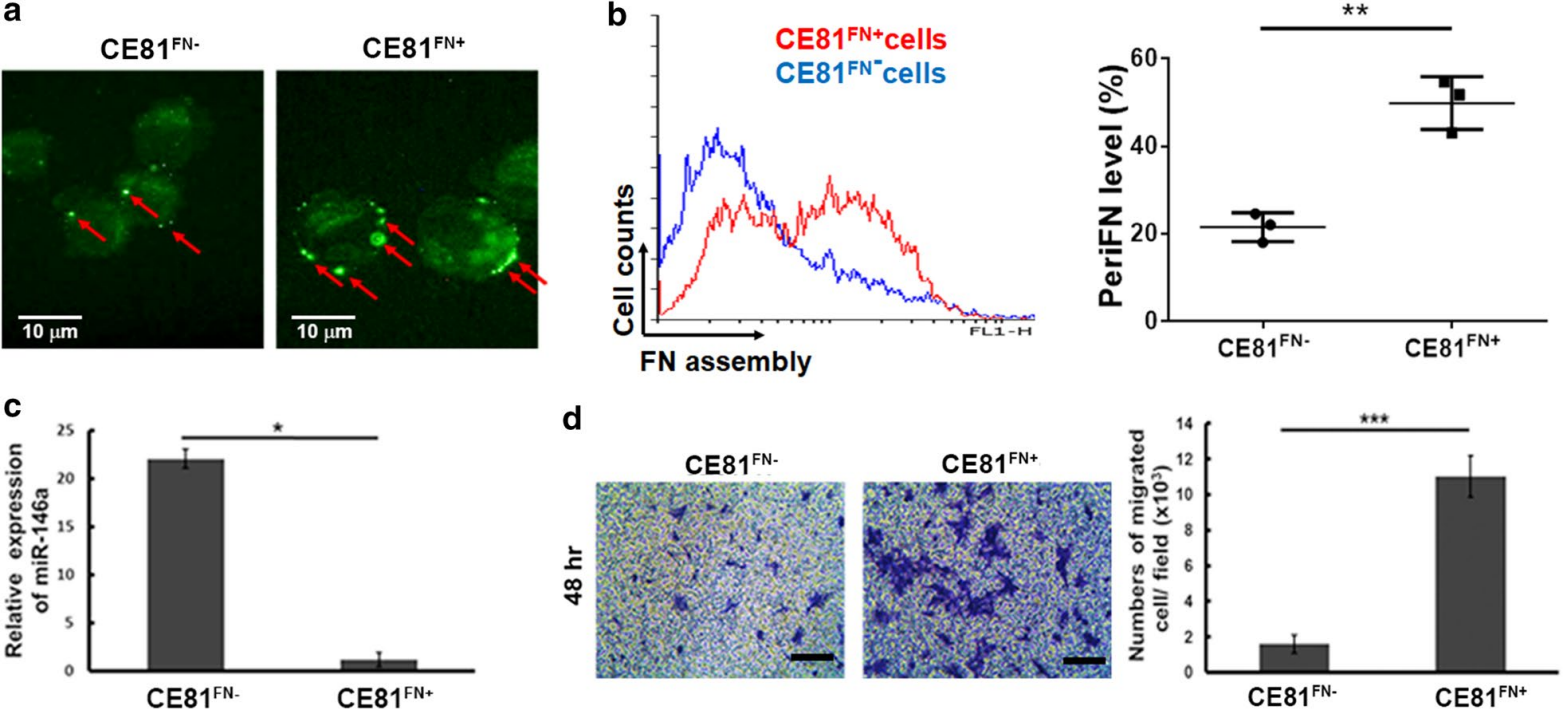
Fibronectin assembly on the membrane correlates with *miR-146a* expression and ESCC cell migration. **a** Fibronectin assembling on cell membrane was shown by FITC conjugated anti-fibronectin antibody under fluorescent microscope (×400, red arrow). **b** The fluorescence signals from CE81^FN−^ and CE81^FN+^ cells merged by flow cytometry software. The pericellular FN (periFN) in CE81^FN−^ and CE81^FN+^ cells were quantified and represented as indicated. **c** The expression level of *miR-146a* in CE81^FN−^ and CE81^FN+^ cells were evaluated by real-time PCR. The data were analyzed by ΔCT method and the relative expression fold was normalized to the internal control U54 gene expression. **d** The migration ability of CE81^FN−^ and CE81^FN+^ cells was investigated using Transwell™ migration assay at 48 h after seeding of cells (10^6^ cells/well). Scale bar: 100 μm. The migrated cell number was counted by Image J software. P values were obtained by Student’s t. test. Statistically significant difference was indicated (*P < 0.05, ***P < 0.001)

Vascular arrest and metastasis of the circulating tumor cells in the lungs could be mediated by the binding between polymeric FN assembled on the surface of tumor cells and endothelial dipeptidyl peptidase IV (DPP IV) [10]. Therefore, we sorted out the ESCC CE81T cells into two groups by flow cytometry according to the expression level of FN that was observed, i.e., high FN assembly on the cell surfaces (CE81^FN+^ cells) and the other with low FN assembly (CE81^FN−^ cells) (Fig. 1a, b). Figure 1a demonstrated that the abundant FN assembly on the membrane of CE81^FN+^ cells compared to that on CE81^FN−^ cells was shown by immunofluorescence staining (Fig. 1a, arrow). Figure 1b indicated that the fluorescence signals from CE81FN- and CE81FN + cells merged and quantified by flow cytometry. Next, we investigated the possibility that miRNAs participate in FN assembly-related cell migration. Therefore, we conducted the miRNA microarray screening. The miRNA expression profile showed that *miR-146a* was the most down-regulated miRNA in CE81^FN+^ cells compared to CE81^FN−^ cells (Table 1, 5.02 fold). Similarly, the expression level of *miR-146a* was significantly lower in CE81^FN+^ cells than in CE81^FN−^ cells as confirmed by real-time PCR (Fig. 1c). We then measured the migration ability of these two cell lines by Transwell™ migration assay at 48 h post-seeding. CE81^FN+^ cells showed a significantly higher migration rate compared to CE81^FN−^ cells (Fig. 1d). In summary, abundant FN assembly on cell membranes was associated with low *miR-146a* expression and high ESCC cell migration.

**Table 1:** Differentially expressed miRNAs in CE81^FN+^ cells compared to CE81^FN−^ cells

Hsa-mir-ID	*P*-value	Fold change^a^	Regulation (FN-P/FN-N)^b^
Hsa-*miR-99a*	0.01	2.16	Up
Hsa-*miR-921*	0.04	1.88	Up
Hsa-*miR-320b*	0.00	1.60	Up
Hsa-*miR-320c*	0.00	1.60	Up
Hsa-*miR-1268*	0.02	1.59	Up
Hsa-*miR-146a*	0.02	5.02	Down
Hsa-*miR-301a*	0.04	1.83	Down
Hsa-*miR-141*	0.03	1.82	Down
Hsa-*miR-345*	0.01	1.58	Down
Hsa-*miR-660*	0.02	1.55	Down

Total RNA extracted from CE81^FN+^ and CE81^FN−^ cells was screened by the miRNA-microarray, which contains 932 probes. The microarray data was analyzed by Biomedical Engineering Center, Industrial Technology Research Institute, Hsintsu, Taiwan. All the *P* values were less than 0.05

^a^The Fold change of CE81^FN+^ vs. CE81^FN−^ is the log ratio value of real-time PCR analysis

^b^FN-P/FN-N represent as FN-Positive and FN-Negative expression CE81T cells

### Prediction and validation of vimentin as a target of *miR-146a* gene in ESCC cells

**Fig. 2:**
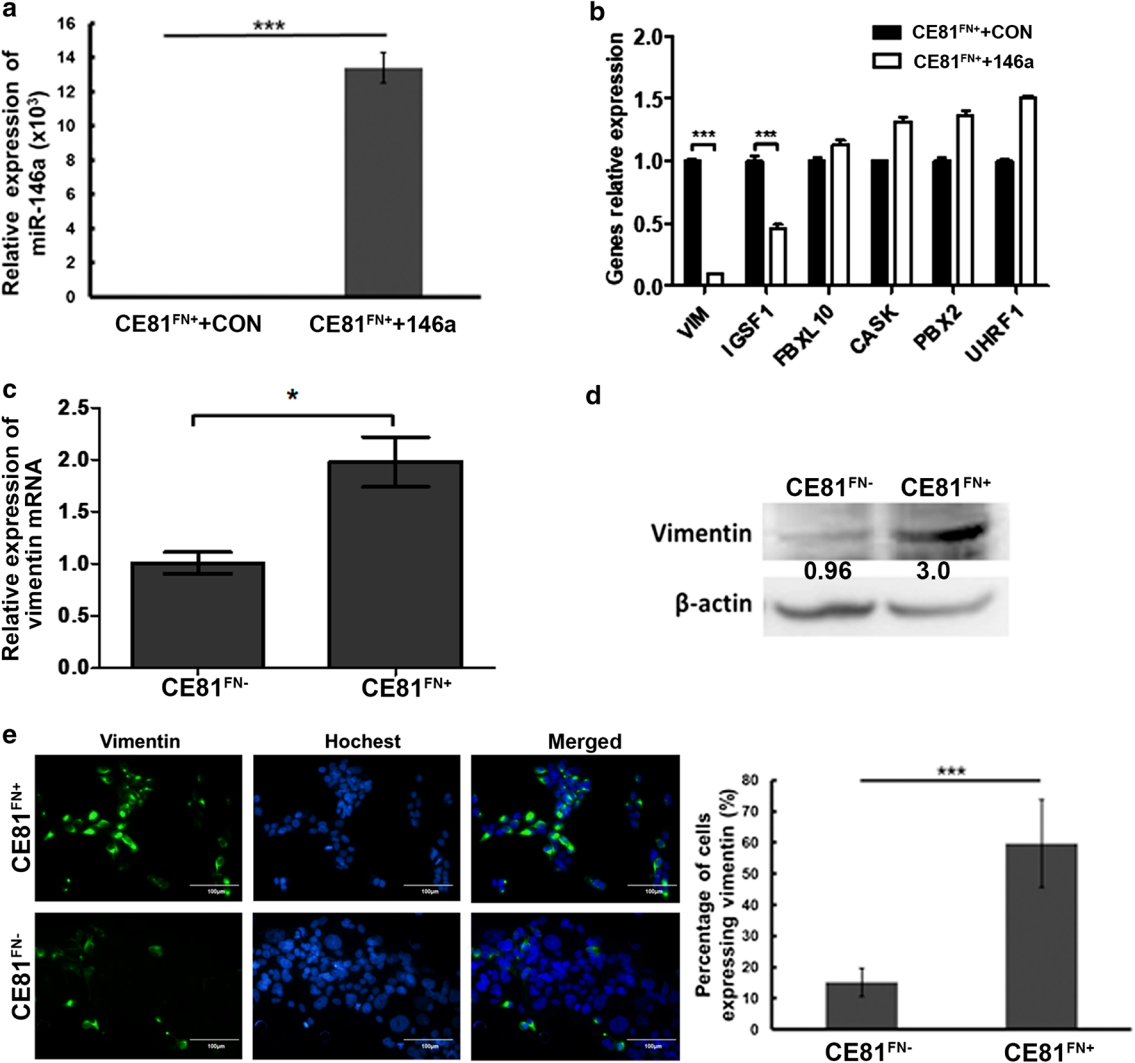
Predication and validation of vimentin as a target of *MiR-146a* gene in ESCC cells. **a** The expression level of *miR-146a* in CE81^FN+^ + CON and CE81^FN+^ + 146a stable cells was evaluated by real-time PCR. The data was analyzed by ΔCT method and the relative expression was normalized to the internal control U54 gene expression. **b** The expression levels of the six overlapped target genes of *miR-146a* predicted by three software programs, i.e., Targetscan, Microcosm and miRNAMap were measured in CE81^FN+^CON and CE81^FN+^146a stable cell lines by real-time PCR. **c** The mRNA expression levels of vimentin in CE81^FN+^ and CE81^FN−^ cells were determined by RT-PCR. **d** The protein levels of vimentin in CE81^FN+^ and CE81^FN−^ cells were determined by Western blotting. β-actin was used as the internal control. **e** Vimentin was labelled by FITC conjugated anti-vimentin antibody in CE81^FN+^ and CE81^FN−^ cells by IFA assay. The cells with fluorescent vimentin expression were shown and quantified by the fluorescent microscopy (×400). Scale bar: 100 μm. P values were obtained from the Student’s t test. Statistically significant difference was indicated (*P < 0.05, ***P < 0.001)

To identify the potential target gene of *miR-146a* involved in ESCC cell motility, we established the stable cell lines CE81^FN+^ + 146a and CE81^FN+^ + CON harboring either *miR-146a* or control miRNA from the parental CE81^FN+^ cells. We confirmed that *miR-146a* was highly expressed in CE81^FN+^ + 146a cells by real-time PCR (Fig. 2a). CE81^FN+^ + 146a cells showed decreased cell proliferation, migration, and invasion compared to the control CE81^FN+^ + CON cells (Additional file 1: Figure S1). We compared the gene expression profiles of CE81^FN+^ + 146a and CE81^FN+^ + CON cells by Illumina™ oligonucleotide microarray analysis. A total of forty most up and down expressed mRNAs were identified (Table 2). We then conducted target gene prediction analysis of these differently expressed genes using three software programs: Targetscan, Microcosm, and miRNAMap. Six overlapped target genes were identified (VIM, IGSF1, FBXL10, CASK, PBX2, and UHRF1). Among these six genes, only vimentin and IGSF gene expression showed an inverse correlation with *miR-146a* level by real-time PCR (Fig. 2b). Vimentin was selected as the target gene of *miR-146a* because it is the only predicated gene down-regulated in CE81^FN+^ + 146a cells detected by the oligonucleotide microarray analysis (Table2, − 0.41). We validated the expression levels of vimentin in CE81^FN+^ and CE81^FN−^ cells, and the results revealed that both mRNA and protein levels of vimentin were significantly higher in CE81^FN+^ cells than in CE81^FN−^ cells by RT-PCR and Western blotting, respectively (Fig. 2c, d). Similarly, high vimentin expression in CE81^FN+^ than in CE81^FN−^ cells was detected by immunofluorescence staining (Fig. 2e, green). In summary, fibronectin assembly-related *miR-146a* is negatively correlated with vimentin protein expression in ESCC CE81 cells.

**Table 2:** The most up and down-regulated mRNA expression profile of ESCC cell lines by oligonucleotide microarray analysis

Gene symbol	Regulation	log FC^a^
NDRG1	Up	4.07
SLC2A3	Up	3.70
SMG1	Up	2.54
IL1A	Up	2.51
LIMCH1	Up	2.51
TMEM123	Up	2.33
DDX21	Up	2.27
HERC5	Up	2.25
NUDT21	Up	2.25
PLOD2	Up	2.25
BIRC3	Up	2.23
IFIT2	Up	2.21
SMC3	Up	2.13
FAM60A	Up	2.08
UBE2V2	Up	2.07
ITGAV	Up	2.05
FAM18B	Up	2.04
CETN3	Up	2.04
ITGB1	Up	2.01
NMD3	Up	1.99
HSPA1A	Down	− 1.94
ATF5	Down	− 1.77
PTHLH	Down	− 1.66
HSPA1B	Down	− 1.62
C16ORF53	Down	− 1.61
GRWD1	Down	− 1.59
MPDU1	Down	− 1.56
TRPM2	Down	− 1.56
DDX54	Down	− 1.55
PTHLH	Down	− 1.51
S100A2	Down	− 1.47
LFNG	Down	− 1.47
MEPCE	Down	− 1.44
DDX39	Down	− 1.43
AVEN	Down	− 1.41
MPDU1	Down	− 1.40
POLR2L	Down	− 1.39
LYPD1	Down	− 1.38
LANCL2	Down	− 1.38
VIM^b^	Down	− 0.41

1—CE81^FN+^ cells were transiently transfected with N.C. or mimic-*miR-146a*. 2—CE81^FN+^ + CON and CE81^FN+^ + 146a stable cell lines. The cells from 1 and 2 were under the microarray analyzed by Illumina gene expression system. The Illumina chip contains 47,231 probes and over 31,000 genes. The data shows the differential gene expression profile of 1 and 2

^a^Represents as log of fold change of 2

^b^Vimentin (VIM) expression was down-regulated in this microarray

### The protein level of vimentin was suppressed in CE81^FN+^ + 146a cells

**Fig. 3:**
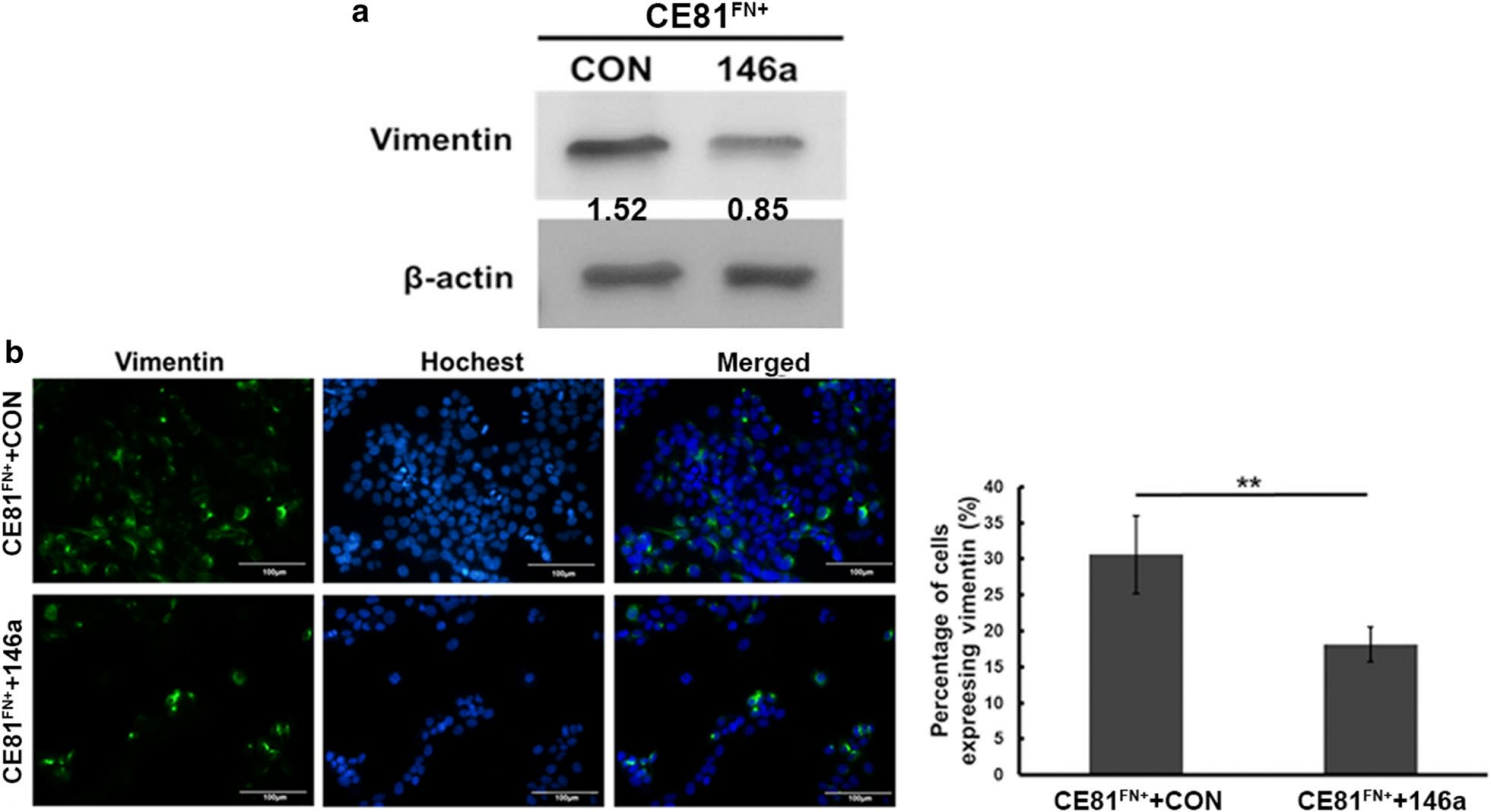
The level of vimentin protein was suppressed in CE81^FN+^146a cells. **a** The protein expression levels of vimentin in CE81^FN+^ + CON and CE81^FN+^ + 146a cells were shown by Western blot analysis. β-actin was used as the internal control. **b** The fluorescence labelled vimentin in CE81^FN+^ + CON and CE81^FN+^ + 146a cells were shown and quantified under a fluorescent microscope. Scale bar: 100 μm. The data were analyzed by Student’s t test. Statistically significant difference was indicated (**P < 0.01)

We compared the protein level of vimentin in the two stable cell lines CE81^FN+^ + 146a and CE81^FN+^ + CON. Accordingly, the vimentin protein level was low at CE81^FN+^ + 146a cells in compared to CE81^FN+^CON cells by Western blotting (Fig. 3a) as well as by immunofluorescence staining (Fig. 3b, green). We constructed the pMIR-luciferase reporter plasmid harboring either the wild-type or mutant vimentin 3′UTR. The luciferase activity was assessed to confirm *miR-146a* targeting vimentin 3′UTR. HEK293T cells were co-transfected with either wild-type or mutant-type vimentin 3′UTR plasmid and pre-*miR-146a* or control microRNA (N.C.) for 48 h (Additional file 1: Figure S2). Our data showed that overexpression of *miR-146a* resulted in significant suppression of wild-type vimentin luciferase reporter activity compared to the control microRNA (N.C.). However, *miR-146a* showed no significant suppression of the mutant vimentin luciferase reporter activity (Additional file 1: Figure S2). Furthermore, human breast cancer cells: MDA-MB-231 ^FN+^/MCF7 ^FN+^ [21] as well as lung cancer cells: CL1-5 ^FN+^/CL1-0 ^FN+^ [11] showing higher migration of FN + cells. In addition, we transiently transfected two ESCC cell lines KYSE 150 (high *miR-146a* expression) and KYSE 70 (low *miR-146a* expression) (Additional file 1: Figure S3A) with anti-*miR-146a* or *miR-146a*, respectively, and evaluated the level of vimentin protein (Additional file 1: Figure S3B and S3C) and cell invasion capability (Additional file 1: Figure S3D). Altogether, these data together with the results in Fig. 2 imply that fibronectin assembly mediated *miR-146a* negative regulation of vimentin expression is a general event in various cancer cells.

### *MiR-146a* inhibited ESCC cell invasion by targeting vimentin

**Fig. 4:**
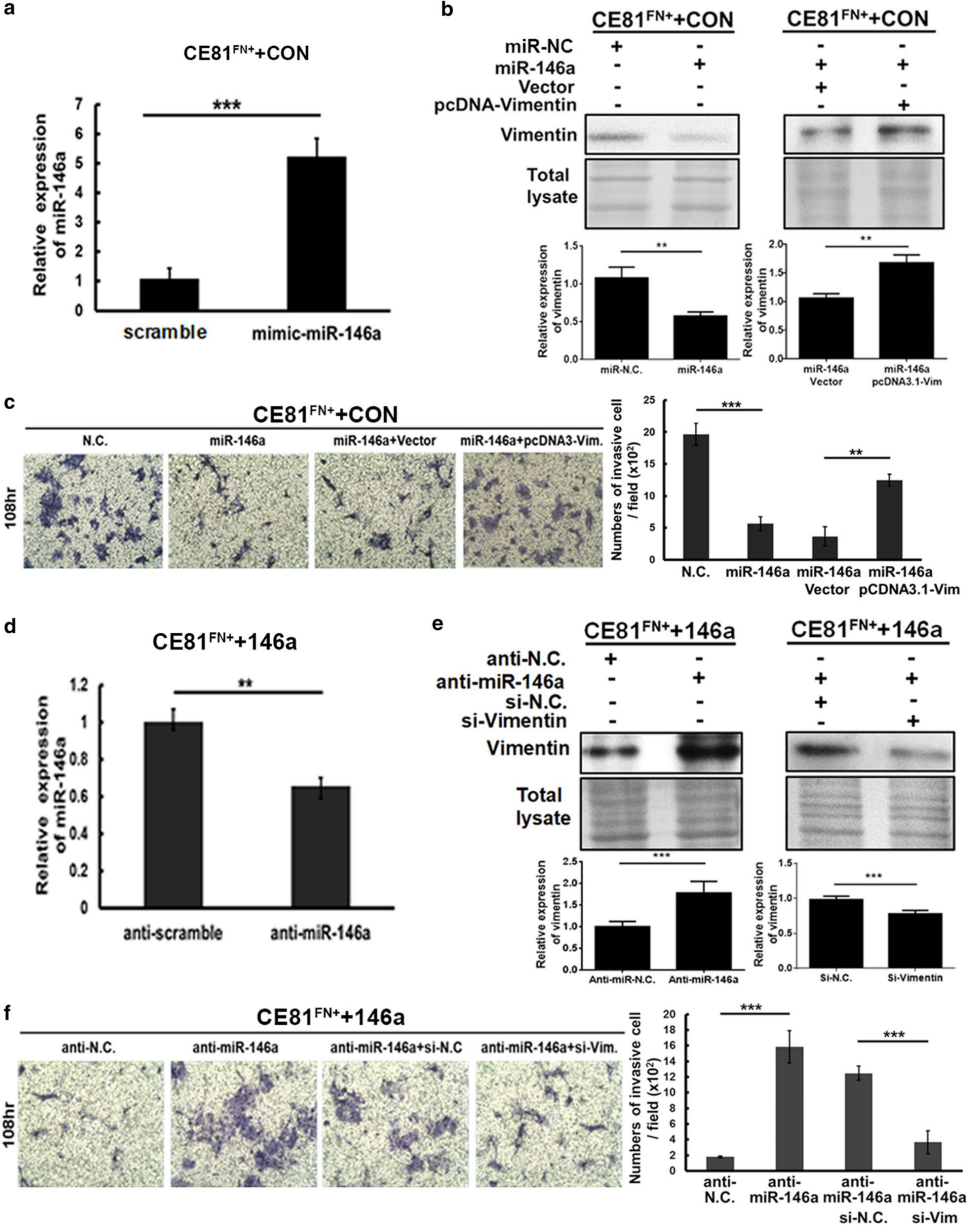
*MiR-146a* inhibited ESCC cell invasion by targeting vimentin. **a** CE81^FN+^ + CON stable cells were transiently transfected with 100 pmol/l of mimic-*miR-146a* or scramble microRNA (N.C.) using Lipofectamine™ for 48 h followed by measuring the expression of *miR-146a* by real-time PCR. **b** CE81^FN+^CON cells harboring transiently transfected scramble (N.C.) or *miR-146a* used in (**a**) were further transiently transfected with pcDNA3.1-vimentin or vector, followed by measuring the level of vimentin using Western blotting. Total lysates in the same blotted PVDF membrane stained by Coomassie blue were used as the internal control. **c** The invasive ability of the cells used in (**b**) was evaluated by Transwell™ invasion assay. **d** CE81^FN+^ + 146a stable cells were transiently transfected with anti-*miR-146a* or anti-scramble microRNA (anti-N.C.) followed by measuring the expression of *miR-146a*. **e** In parallel, the study was CE81^FN+^*miR-146a* cells harboring anti-scramble (anti-N.C.) or anti-*miR-146a* were further transfected with si-Vimentin or si-RNA negative control (si–N.C.). The levels of vimentin and β-actin were investigated by Western blotting. Total lysates in the same blotted PVDF membrane stained by Coomassie blue were used as the internal control. **f** The invasive abilities of the cells used in (**e**) were measured by Transwell™ invasion assay. The numbers of the invaded cells on the bottom of the membrane were counted after incubating for 108 h. The quantitative data are shown. P values were obtained by Student’s t test, **P < 0.01, ***P < 0.001. The quantifications for (**b**) and (**e)** were derived from at least three independent biological repetitions (Additional file 1: Figure S7)

To confirmed the role of *miR-146a* together with its negatively regulated vimentin in the tumorigenesis of ESCC cells, the CE81^FN+^ + CON stable cell line shown in Fig. 3 was transiently transfected with scramble microRNA or mimic-*miR-146a*, and *miR-146a* overexpression was confirmed by real-time PCR (Fig. 4a). Accordingly, we detected decreased protein level of vimentin (Fig. 4b, left panel), accompanied with decreased invasion capability of the CE81^FN+^ + CON cells overexpressing mimic-*miR-146a* (Fig. 4c, miR-146a vs. N.C.). To verify the relationship between vimentin and *miR-146a* as well as their effects on cell motility, we showed that the protein level of vimentin increased in CE81^FN+^ + CON cells co-transfected with mimic-*miR-146a* and pcDNA3-vimentin (Fig. 4b, right panel), accompanied by increased cell invasion compared to the vector control (Fig. 4c, miR-146a + pcDNA3.1-Vim vs. miR-146a + Vector).

Similarly, we transiently introduced anti-scramble microRNA (anti-N.C.), and anti-*miR-146a* into CE81^FN+^ + 146a stable cells as shown in Fig. 4d. We found that the endogenous *miR-146a* level was significantly decreased (> 35%) in CE81^FN+^ + 146a cells with *anti-miR-146a* compared to the CE81^FN+^ + 146a cells with anti-scramble *miRNA* (Fig. 4d). The level of vimentin was increased in CE81^FN+^ + 146a cells harboring anti-*miR-146a* compared to the scramble anti-N.C. group (Fig. 4e, left panel). Accordingly, the invasion of CE81^FN+^ + 146a cells transiently expressing anti-*miR-146a* was significantly higher compared to the anti-N.C. group (Fig. 4f, anti-*miR-146a* vs. anti-N.C.). We further silenced the expression of vimentin in the CE81^FN+^ + 146a + anti-miR 146a cells with a small interfering RNA of vimentin (si-Vim) (Fig. 4e, right panel). Consequently, the invasion of the CE81^FN+^ + 146a + anti-*miR-146a* + si-vimentin cells was significantly decreased compared to the CE81^FN+^ + 146a + anti-*miR-146a* + si–N.C. cells (Fig. 4f, anti-miR-146a + si-vimentin vs. anti-miR-146a + si–N.C.). The negative regulation between *miR-146a* and vimentin was further confirmed using HEK293T cells due to its high transfection efficiency (Additional file 1: Figure S4). Similar results were observed in another two ESCC cell lines as shown in Additional file 1: Figure S2. Taken together, above data clearly imply that *miR-146a* suppresses cell invasion through downregulation of vimentin expression in various ESCC cells.

### *MiR–146a* and vimentin expression levels correlated with ESCC tumor stage and overall survival rate of ESCC patients

**Fig. 5:**
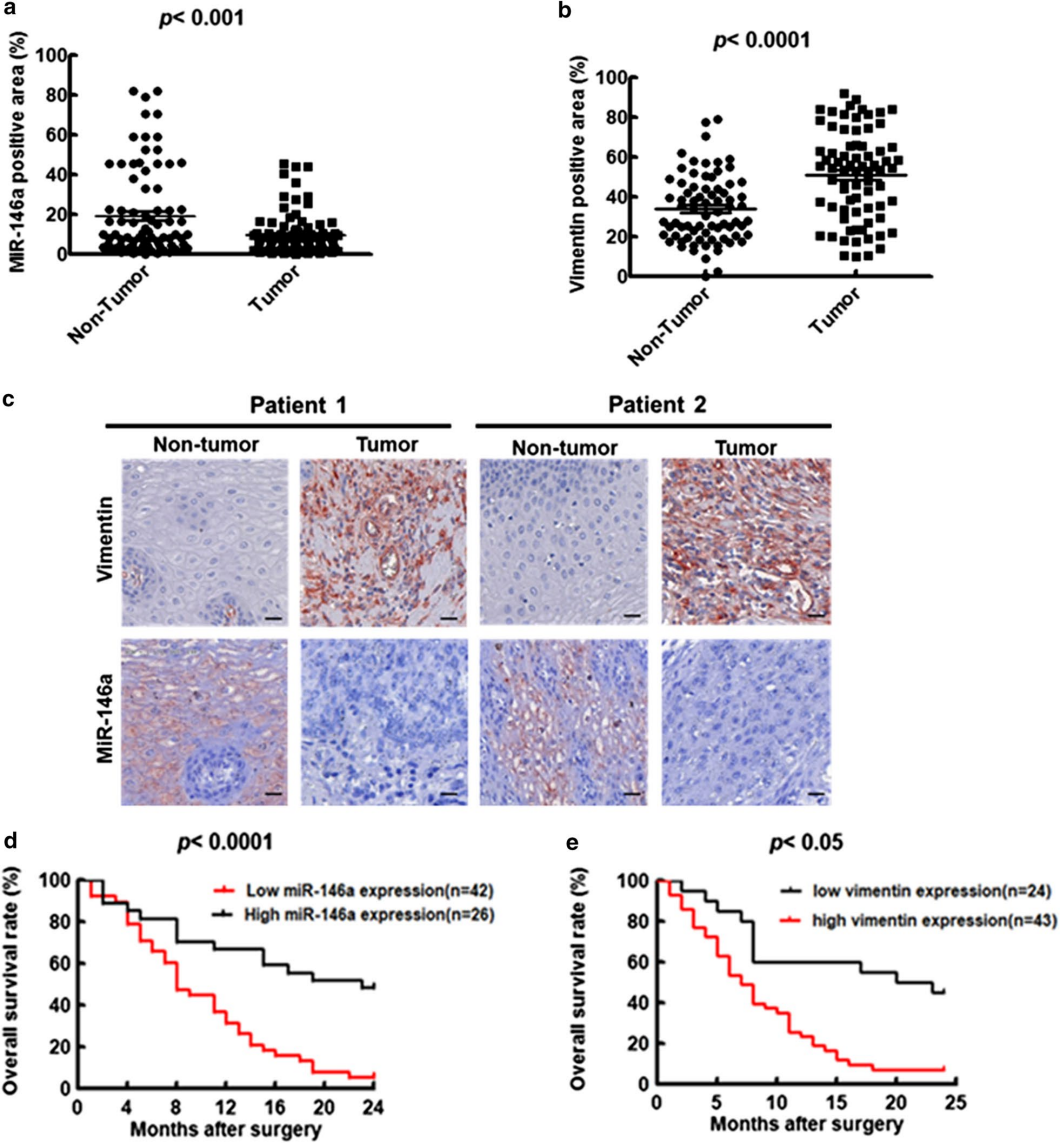
Low *miR-146a* or high vimentin expression levels in the tumorous ESCC tissue array correlated with poor overall survival rate. **a** The *miR-146a* levels in the tumorous and adjacent non-tumorous cells of 68 paired ESCC patient specimens in the tissue array after ISH treatment were quantified by defining regions of interest (ROI) using automated cell acquisition and quantification software (Histoquest™) (P < 0.001). **b** The protein levels of vimentin in the same ESCC specimens used in (**a**) were stained by IHC followed by Histoquest™ quantification (P < 0.0001). **c** Two representative patient specimens containing tumorous and adjacent non-tumorous cells from the 68 ESCC patients were analyzed to determine *miR-146a* and vimentin expression levels after ISH and IHC treatment. Hematoxylin was used to stain nucleus and AEC (3-amino-9-ethylcarbazole) for *miR-146a* and vimentin expression. Scale bar 20 μm. The correlation of (**d**) *miR-146a* level (P < 0.0001) and (**e**) vimentin expression (P < 0.05) with ESCC patient overall survival rate were assessed by Kaplan–meier analysis and Log rank test

To clarify the significance of *miR-146a* and the target gene vimentin in clinical ESCC patients, two groups of ESCC patient specimens were analyzed (Table 3). The first set of 68 ESCC patient specimens (Group I, Table 3) was used for measuring *miR-146a* level by real-time PCR. The *miR-146a* level was significantly higher in the early ESCC tumor stages (I + II) compared to the late tumor stages (III + IV) (Additional file 1: Figure S5). The expression levels of *miR-146a* and vimentin in the second set of 68 paired ESCC patient specimens (non-tumor vs. tumor) in the tissue array (Group II, Table 3) were measured by ISH and IHC staining. The level of *miR-146a* was significantly lower in tumorous ESCC compared to non-tumorous ESCC by ISH staining (Fig. 5a). In contrast, vimentin protein level was significantly higher in tumorous cells compared to non-tumorous cells by IHC staining (Fig. 5b). The expressions of *miR-146a* and vimentin in tissue sections of two representative ESCC patients were evaluated by ISH and IHC staining, respectively. We detected low *miR-146a* and high vimentin expression in the tumorous cells of these two representative patients (Fig. 5c), which were consistent with the results presented above. Furthermore, the associations of *miR-146a* and vimentin with overall survival rate among these ESCC patients were analyzed by Kaplan–Meier analysis and log rank test. Our data revealed that the poor overall survival rate of ESCC patients 2 years after surgery were significantly correlated with either low *miR-146a* expression (cut off value: < 0.8) or high vimentin expression (cut off value: > 1.8) but not with *miR-146a* + vimentin (Fig. 5d, e and Additional file 1: Table S2). We conducted the clinicopathologic parameter analysis of *miR-146a,* vimentin and *miR-146a* + vimentin using sixty-eight ESCC patients (Table 3, Group II). No further correlation of *miR-146a,* vimentin and *miR-146a* + vimentin with age and gender was detected (Additional file 1: Table S2). Above data imply that the negative correlation between *miR-146a* and vimentin affects ESCC tumorigenesis and tumor progression, both in vitro and in ESCC patient specimens.

**Table 3:** Characteristics of Group I and Group II of ESCC clinical specimens

Characteristics	Group I (ESCC patient specimens)	Group II (non-tumor vs. tumor)
Patients no	68	68
Age (years)		
Median age	57	57
Range	36–87	34–82
Sex		
Male/female	64/4	65/3
Tumor stages		
I	8	6
II	19	14
III	31	43
IV	10	5
Two-year survival		
≥ 24 months	32	18
< 24 months	36	50

Group I is the characteristics of ESCC patients for measuring the miR-146a RNA expression levels by real-time RT-PCR

Group II is the characteristics of ESCC patient specimens (non-tumor vs. tumor) for direct detection of *miR-146a* and vimentin expression levels by ISH and IHC staining

## Discussion

In this study, we demonstrated that the level of membrane-bound FN was negatively correlated with the expression of *miR-146a* which suppressed ESCC cell motility by targeting vimentin, a marker of EMT during normal development or metastatic progression. We found that the *miR-146a*-vimentin axis is involved in tumor formation, tumor stage and overall survival rate in ESCC patients. This is the first report to reveal that *miR-146a* suppresses migration and invasion through the negative regulation of vimentin in ESCC cells.

Expression of FN that is assembled on the cell membrane as a polymeric form plays a pivotal role in promoting tumor progression, including cell migration, invasion, and cancer metastasis [7, 9, 11]. Indeed, aggregation of FN on the cell membrane is highly-correlated with metastasis in various cancer cell types [10, 22, 23]. FN, a metastatic related gene, is also highly expressed in diverse tumors [23], and modulates multiple functions of tumor cells, including stimulation of cell proliferation, differentiation, and metastatic cascade in melanoma and ovarian cancers via activation of various cell surface receptors such as integrin [22, 24]. Zhang et al*.* reported that FN expression is up-regulated during lung cancer metastasis [25]. Here, we found that FN assembled on the ESCC tumor cell membrane was highly associated with low expression level of *miR-146a*, which led to increased ESCC cell invasion through increased vimentin expression.

Increased of ESCC tumorigenesis through the targeting of PTEN or REPS2 by *miR-21* and *miR-373* have been reported [26, 27]. In contrast, the expression levels of *miR-205* and *miR-375* decreased in tumorous parts of ESCC patient specimens by targeting Zeb and PDK1 genes [28, 29]. The above findings indicate the complexity of miRNAs, their target genes, as well as their functions in ESCC tumorigenesis.

MiRNA plays critical roles in the regulation of tumorigenesis and various signaling pathways including, apoptosis, inflammation and immune responses [30, 31]. *MiR-146* is further classified as *miR-146a* and *miR-146b*, which are located on chromosome 5 and 10, respectively. Only two nucleotides differ between the mature form of *miR-146a* and *miR-146b* at the 3′-end. However, the underlying mechanism involved in the regulation of *miR-146a* and *miR-146b* remains unclear [32]. Katakowski et al. reported that overexpression of miR-146b decreases glioma cell migration and invasion [33]. Moreover, it has also been shown that *miR-146b* is overexpressed in thyroid tumor, colorectal cancer, and melanoma [34]. In lung and pancreatic cancers, *miR-146a* is defined as an anti-tumor RNA, inhibits cell migration and invasion through suppression of EGFR signaling [35, 36]. It also plays a suppressive role in breast, gastric, and prostate cancers In contrast, *miR-146a* functions as an oncogene in melanoma, which promotes tumor initiation and progression by activating Notch signaling [37, 38]. Therefore, *miR-146a* may function either as an oncogene or a tumor suppressor in tumorigenesis depending on the types of tumors. In addition, *miR-146a* negatively regulates the pro-inflammatory chemokine IL-8 and the innate immune response by interfering with the NF-κB pathway [31, 39]. In this study, we demonstrated that *miR-146a* plays a suppressive role in ESCC tumorigenesis and tumor progression, including cell proliferation, colony and tumor formation (data not shown), migration and invasion.

Since downregulation of *miR-146a* in ESCC cells is associated with high FN assembly on cell membranes, it is conceptually possible that both events are causally related. It has been reported that the transcription of *miR-146a* can be activated by NF-κB [40, 41] and activation of AKT is capable of triggering nuclear transportation of NF-κB to become an active transcription factor [42, 43]. Interestingly, we demonstrated that AKT is inactivated in suspended tumor cells with high FN assemble on the cell membrane [8]. These findings imply that FN assembly on tumor cell membrane may cause downregulation of *miR-146a* by suppressing the PI3K/AKT/NF-κB signaling pathway [44]. Furthermore, it has been shown that the metastatic suppressor, BRMS1, increases the level of *miR-146a* in cancer cells to reduce their metastatic potential [45]. Since FN assembly on tumor cell membranes is highly associated with the metastatic potential of tumor cells [9, 11, 46], it is likely that FN assembly decreases BRMS1 to circumvent metastatic suppression by reducing BRMS1-promoted *miR-146a* levels [45]. Furthermore, WWOX, a negative regulator of c-Myc, has been demonstrated to inhibit the FN expression level and metastatic ability of triple-negative breast cancer (TNBC) by upregulating *miR-146a* [47]. In line with these findings, upregulation of SOX5, a target gene of *miR-146a*-5p, and downregulation of *miR-146a*-*5p* have been reported in TNBC [48]. Overexpressing SOX5 significantly eliminates the effects of *miR-146a-5p* mimics in TNBC cells and increases the expression of mesenchymal markers including FN and vimentin [48]. Furthermore, *miR-146a* mimics decrease the TGF-β-induced fibronectin in orbital fibroblasts by lowering Smad4 and TRAF6 protein levels [49], indicating that *miR-146a* may lead to the inhibition of FN expression in ESCC cells. Altogether, these contradictory findings warrant further investigation of the causal relationship between *miR-146a* and FN assembly on ESCC cell membranes.

Guo et al*.* reported that functional SNPs of pre-*miR-146a* contribute to ESCC susceptibility and clinical outcome [50]. Here, we reveal that FN assembly on ESCC cell membrane was associated with decreased expression of *miR-146a*, leading to upregulation of vimentin and promotion of ESCC cell motility. More importantly, emerging evidence indicated that FN expression is highly associated with poor overall survival rates in variety of cancer types such as colorectal and gastric cancer [51, 52]. On the other hand, high expression of *miR-146a* were correlated with prolonged overall survival [53]. Based on our finding, the FN assembly is negative correlation with *miR-146a* regarding to cancer patients overall survival rates. Notably, we demonstrated that either low *miR-146a* or high vimentin expression was significantly correlated with tumor formation, tumor stages and poor overall survival rate of ESCC patients (Fig. 5). These findings may be valuable for developing a novel drug targeting strategy toward *miR-146a* and vimentin for treatment of late stages ESCC.

Vimemtin is the major component of the intermediate filaments and, along with FN, contributes to epithelial-mesenchymal transition (EMT) as well as cancer cell metastasis. Similarly, overexpression of vimentin in ESCC cells is correlated with increased tumor growth, invasion, poor prognosis, and lymph node metastasis [54, 55]. Sudo et al. reported that the high expression of both FN and vimentin was associated with advanced tumor stage and poor prognosis in ESCC patients [14]. Actually, current research suggests that FN promotes EMT in variety of malignant cancer types and is one of well-known biomarkers of EMT for cancer metastasis [7]. Indeed, we found that downregulation of *miR-146a* led to upregulation of vimentin thereby promoting cell proliferation, colony and tumor formation as well as cell motility of ESCC cells that highly assemble FN on the cell membrane.

The discrepancy of the correlation between the expression level of vimentin and the level of cell invasion (Fig. 4) indicates the possibly that *miR-146a* may target other molecules such as IGSF1 (Fig. 2b), which are also required for ESCC cell motility. Nevertheless, this experiment disclosed the trend of a negative correlation between *miR-146a* and vimentin plus cell invasion. More importantly, the underlying mechanism for downregulation of *miR-146a* through epigenetic alteration during ESCC progression might be elucidated in the future. Taken together, from our findings we hypothesize that fibronectin assembly-related low *miR-146a* expression induces ESCC cell mobility through regulation of its targeting gene vimentin (Additional file 1: Figure S6).

*MiRNA-96* decreases cancer cell invasion and migration by inhibition of K-*ras* gene expression and triggering of apoptosis [13]. *MiR-200c* suppresses proliferation by down-regulating mutant K-*ras* expression in breast and lung cancer cells [56]. Moreover in colorectal cancer, *let-7* miRNA suppresses K-*ras* activity and p53 expression [57]. These studies imply that an association exists between various miRNAs and the *ras* gene*.* It has been reported that increased *miR-146a* suppresses the expression of EGFR, ERK1/2 and K-*ras* genes, resulting in the inhibition of cell migration, invasion and proliferation of pancreatic cancer and non-small cell lung cancer cells [35, 58]. The above findings imply that *miR-146a* affects the activity of K-*ras*. However, the regulatory relationship between K-*ras* and *miR-146a* in ESCC warrants further study.

In summary, we identified a new regulatory mechanism whereby *miR-146a* suppresses the cell motility of ESCC cells that highly assemble FN on cell membranes by inhibiting vimentin expression. Our findings warrant further exploration to determine the roles of these two genes in ESCC cell metastasis in vivo.

## Conclusions

ESCC patients in Taiwan are at high risk of developing tumor cell metastasis. However, the underlying mechanism remains unclear. High fibronectin membrane assembly ESCC cells (CE81^FN+^) showed increased migration and invasion compared to low fibronectin membrane assembly cells (CE81^FN−^). *MiR-146a* expression was the most down-regulated miRNA in CE81^FN+^ cells compared to CE81^FN−^ cells. *MiR-146a* expression level was inversely correlated with the mobility of ESCC cells. We identified vimentin as the target of *miR-146a* in ESCC tumorigenesis and mobility, and further analysis confirmed this finding. Analysis of one hundred and thirty-six ESCC patient specimens disclosed that low *miR-146a* expression or high vimentin level was significantly associated with tumor formation and poor overall survival rate. This is the first report to link FN cell membrane assembly with *miR-146a*, vimentin and ESCC tumorigenesis both in vitro and in ESCC patients.

## Supplementary information

**Additional file 1: Figure S1.** Proliferation, migration and invasion of CE81^FN+^ + CON and CE81^FN+^ + 146a cells. **Figure S2.** *Mir-146a* targeting vimentin 3′-UTR was examined by luciferase reporter assay in HEK 293 T cells. **Figure S3.** *MiR-146a* and negatively regulated vimentin affect ESCC KYSE cell migration. **Figure S4.** Protein expression of vimentin is negative regulation by *miR-146a.* **Figure S5**. *MiR-146a* level inversely correlates with ESCC patient tumor stages. **Figure S6**. The hypothetic model of fibronectin assembly-mediated *miR-146a* suppressing ESCC cell mobility through targeting vimentin. **Figure S7.** Raw data of western blot images for Fig. 4B, E in triplicate or quadruplicate. **Table S1.** List of primers and sequences. **Table S2.** Correlation of *miR-146a* and vimentin protein expression with clinicopathologic parameters of sixty-eight ESCC patients

## Abbreviations

ESCC: Esophageal squamous cell carcinoma
FN: Fibronectin
miRNA: MicroRNA
CE81^FN^^+^: ESCC CE81T cells with high-FN assembly
CE81T^FN^^−^: ESCC CE81T cells with low-FN assembly
EMT: Epithelial mesenchymal transition
ECM: Extracellular matrix
RT-PCR: Reverse transcription-polymerase chain reaction

## Availability of data and materials

The datasets used and/or analyzed in the current study are available from the corresponding author on reasonable request.
