# Supplementary material for: MicroRNA-146a suppresses tumor malignancy via targeting vimentin in esophageal squamous cell carcinoma cells with lower fibronectin membrane assembly
Source: J Biomed Sci. 2020 Nov 28;27:102. doi: 10.1186/s12929-020-00693-4 (PMC7697386; doi:10.1186/s12929-020-00693-4)
Supplement: Supplementary file 1 — Additional file 1: Figure S1. Proliferation, migration and invasion of CE81FN+ + CON and CE81FN+ + 146a cells. Figure S2. Mir-146a targeting vimentin 3′-UTR was examined by luciferase reporter assay in HEK 293 T cells. Figure S3. MiR-146a and negatively regulated vimentin affect ESCC KYSE cell migration. Figure S4. Protein expression of vimentin is negative regulation by miR-146a. Figure S5. MiR-146a level inversely correlates with ESCC patient tumor stages. Figure S6. The hypothetic model of fibronectin assembly-mediated miR-146a suppressing ESCC cell mobility through targeting vimentin. Figure S7. Raw data of western blot images for Fig. 4B, E in triplicate or quadruplicate. Table S1. List of primers and sequences. Table S2. Correlation of miR-146a and vimentin protein expression with clinicopathologic parameters of sixty-eight ESCC patients [file 12929_2020_693_MOESM1_ESM.docx]

**Additional data**

**Additional Figure S1**

**
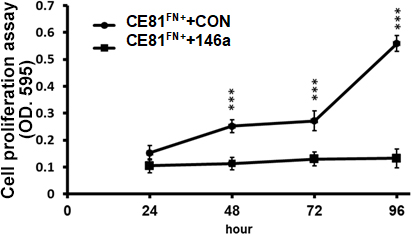
A**

**
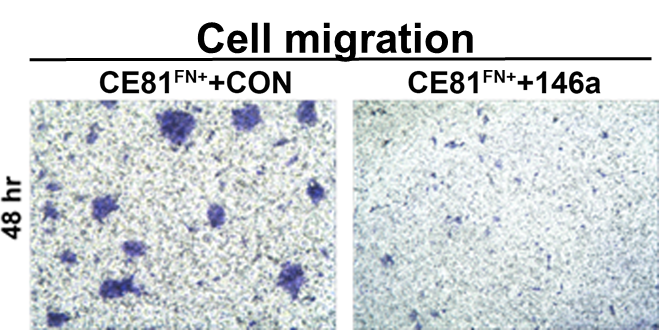
B**

**
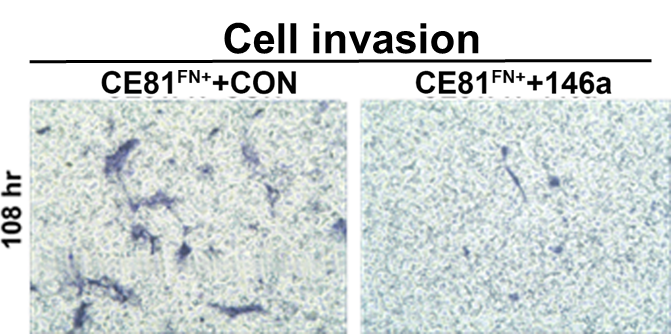
**
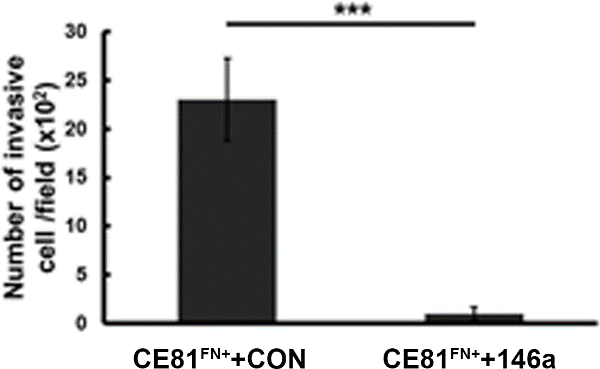
**C**


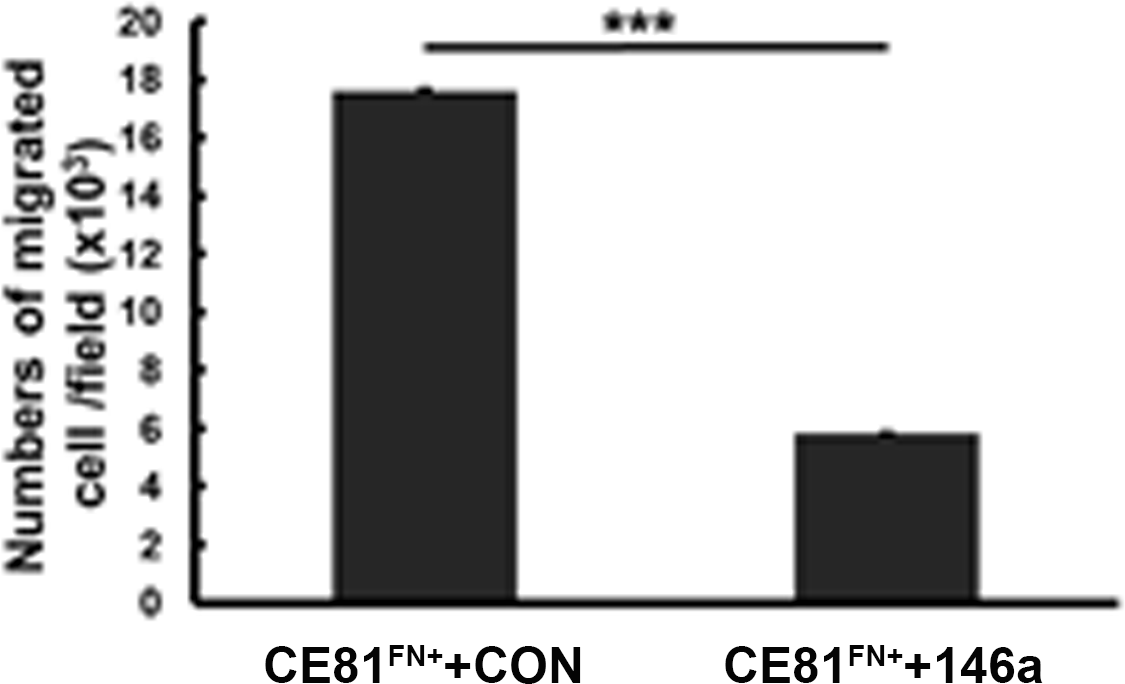


**Additional Figure S2**

**
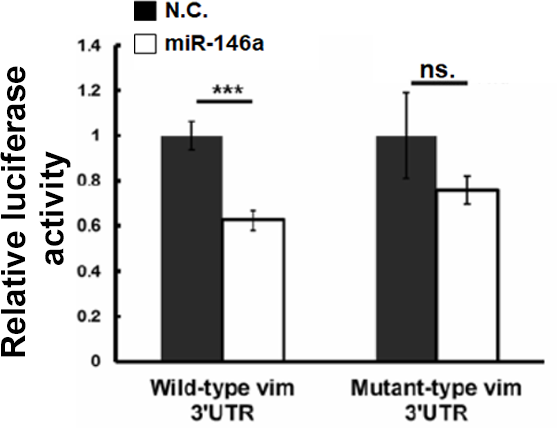
**

**Additional Figure S3**

**
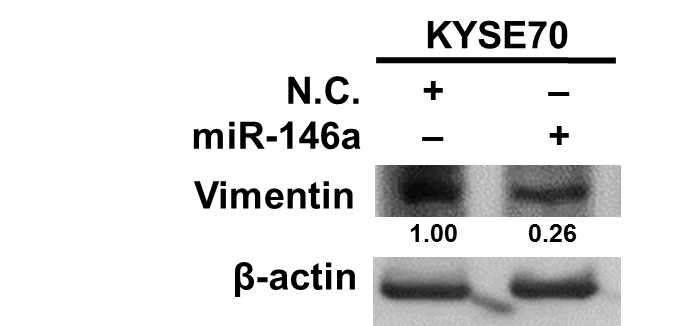

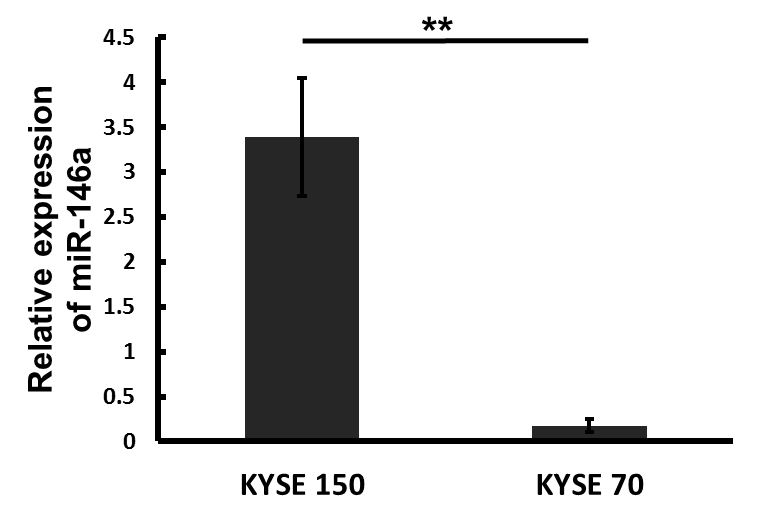
A**

**
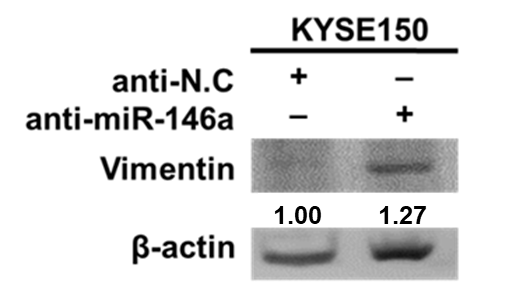
B C**


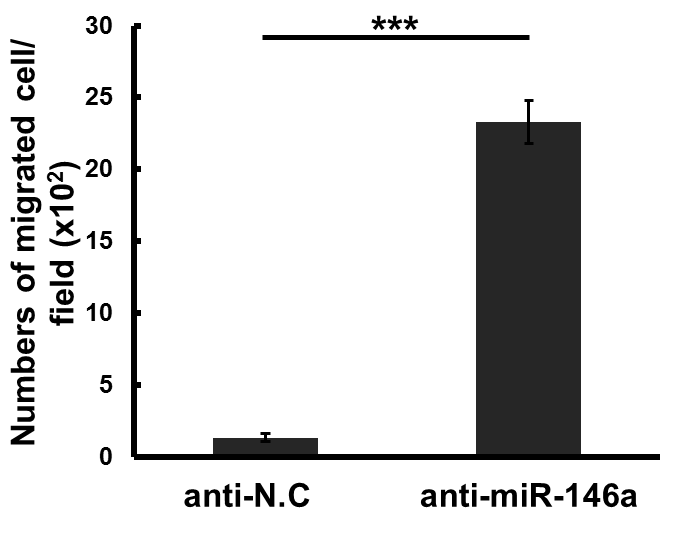

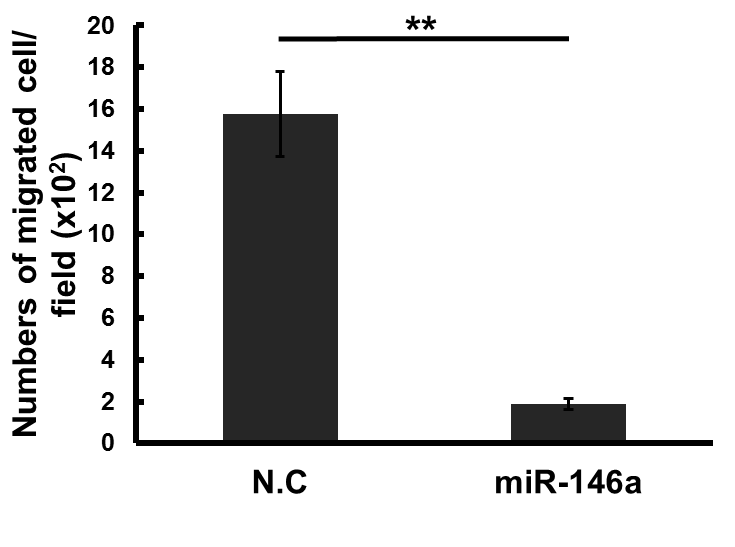
**
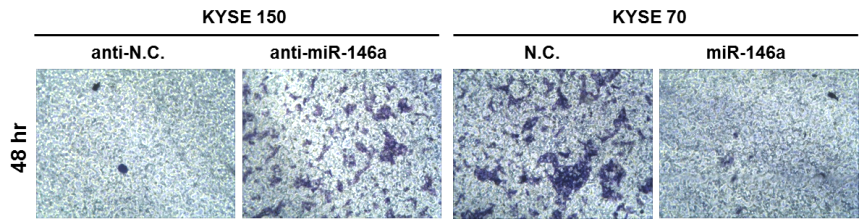
D**

**Additional Figure S4**

**
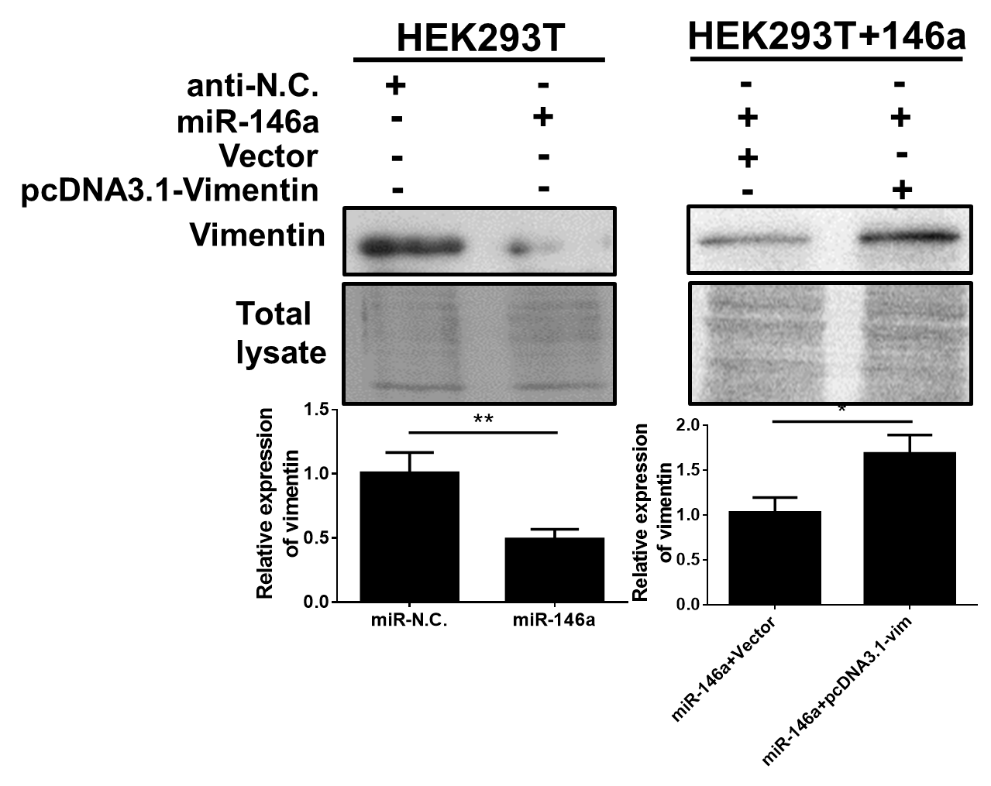
 A**

**
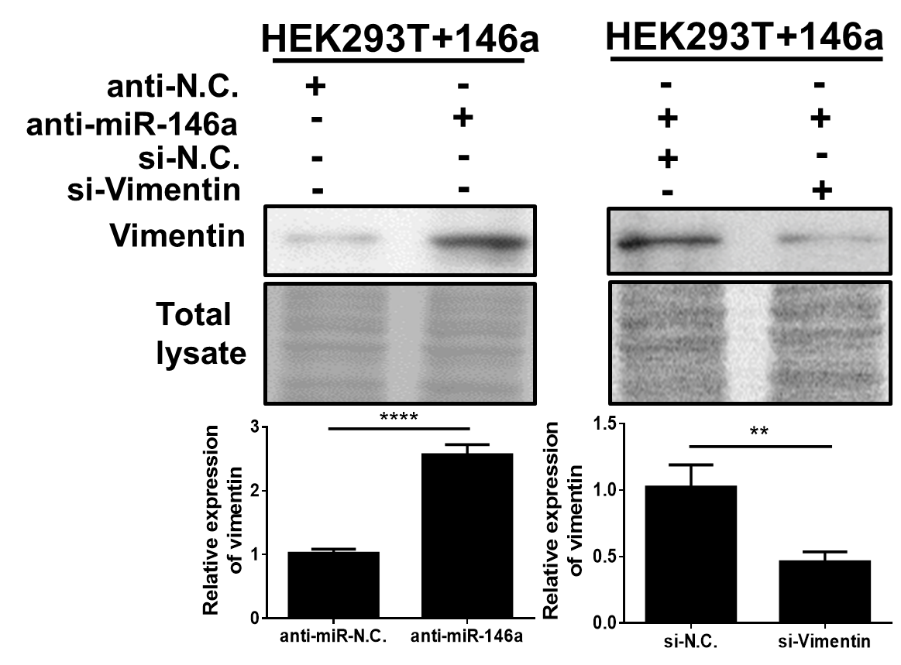
 B**

**Additional Figure S5**

**Additional Figure S6**

**
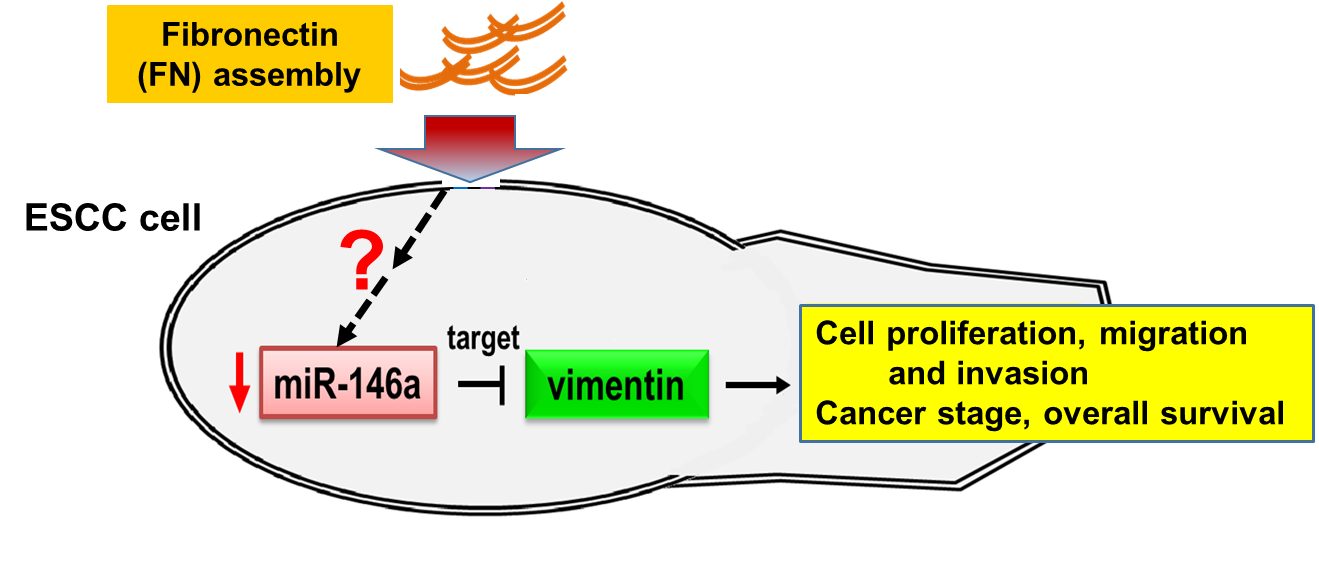
**

**
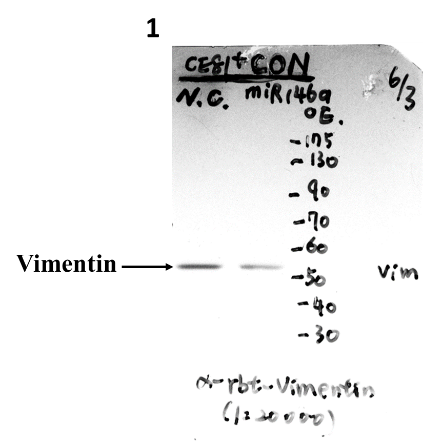
Additional Figure S7**

**
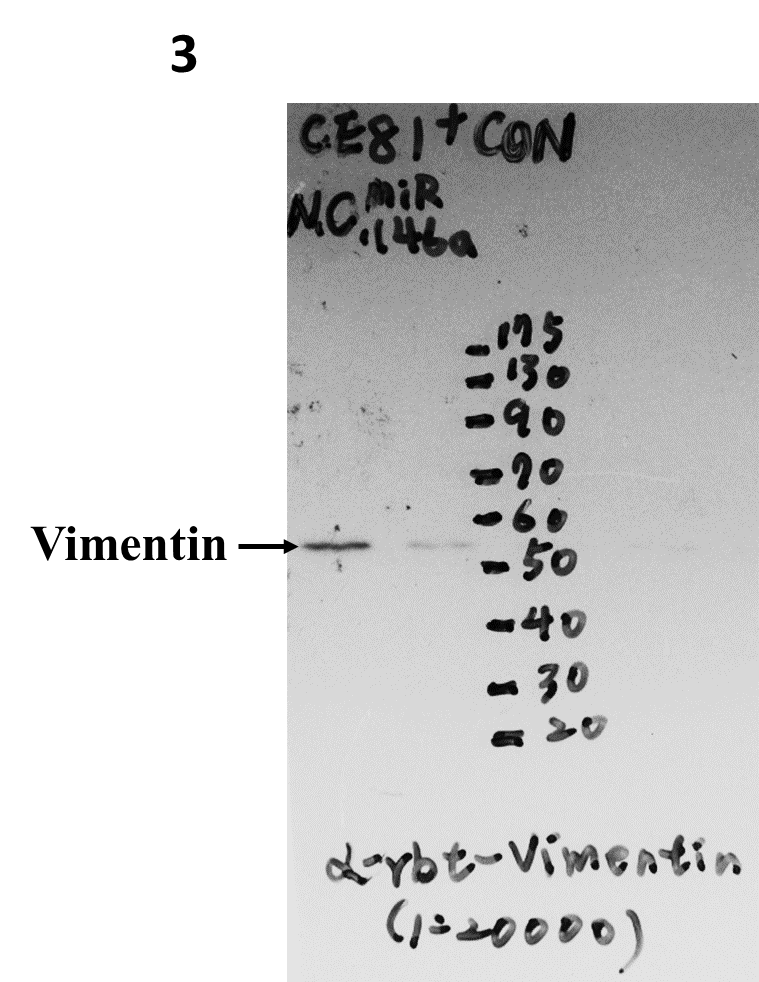

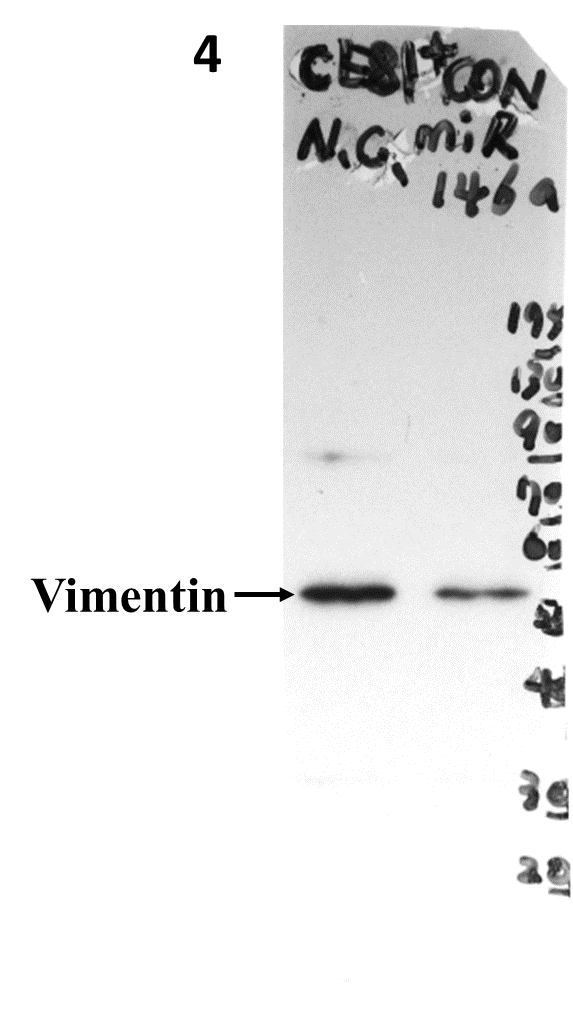

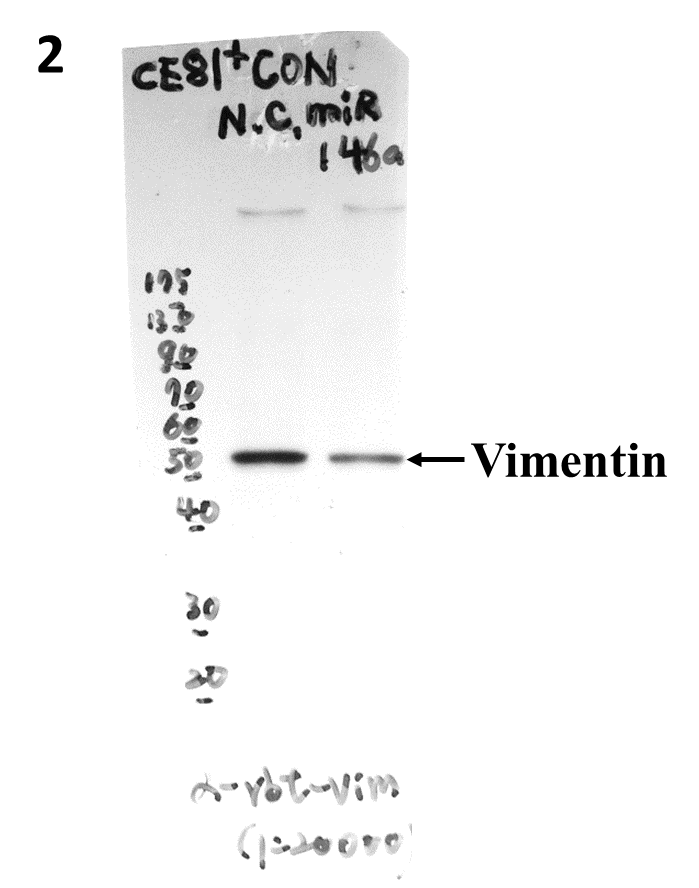
A**

**
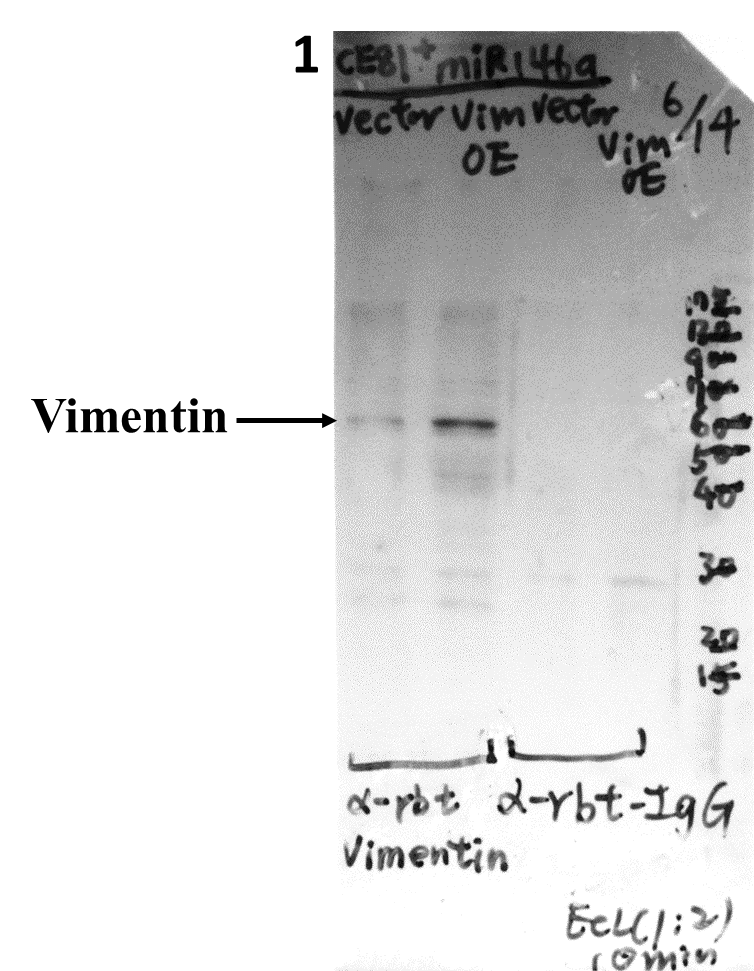

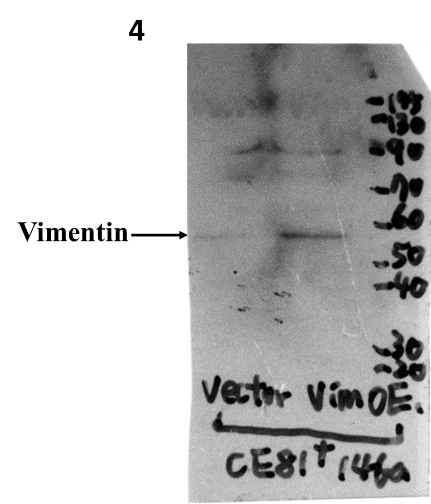

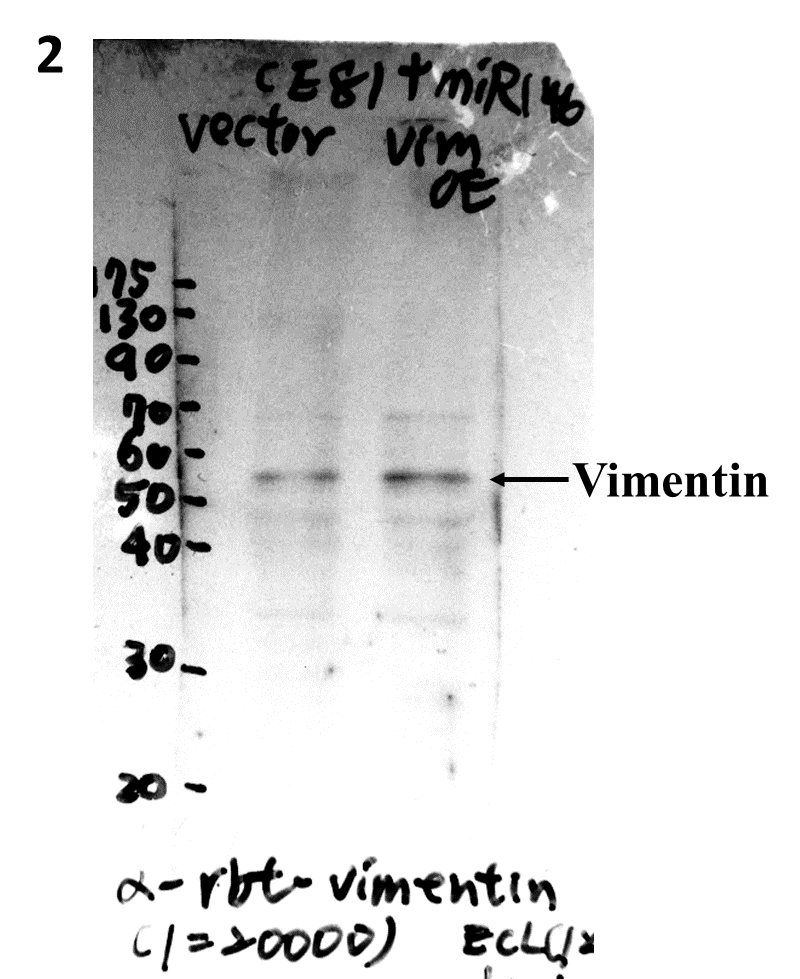

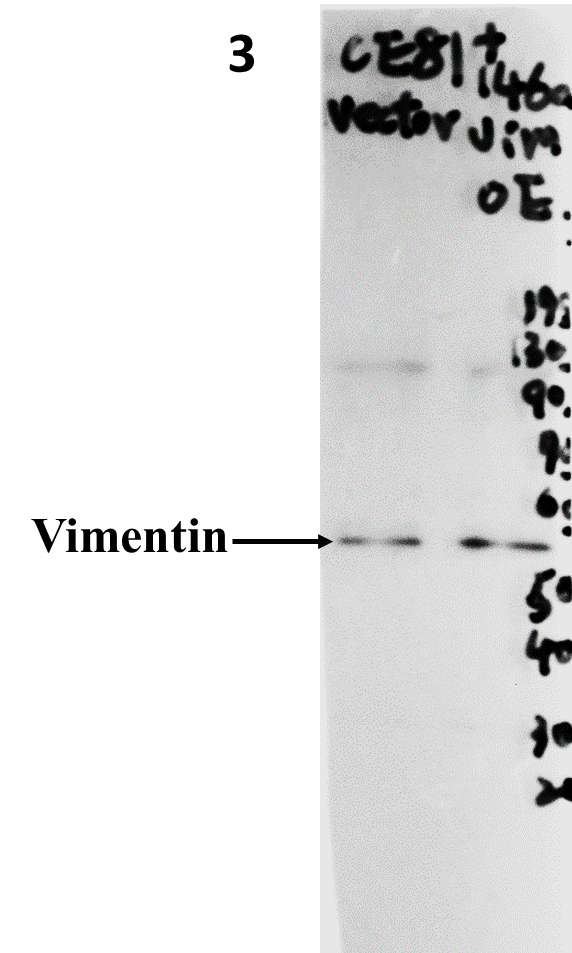
B**

**
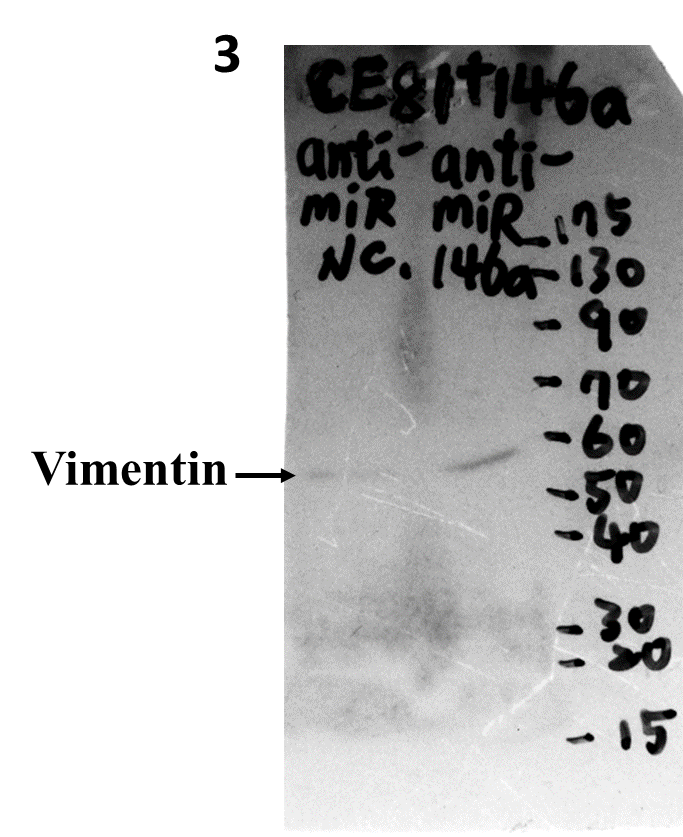

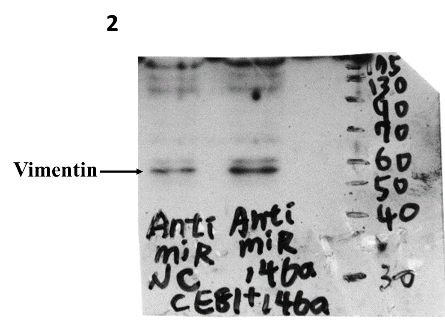

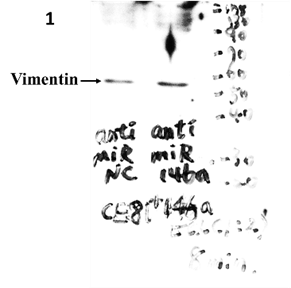

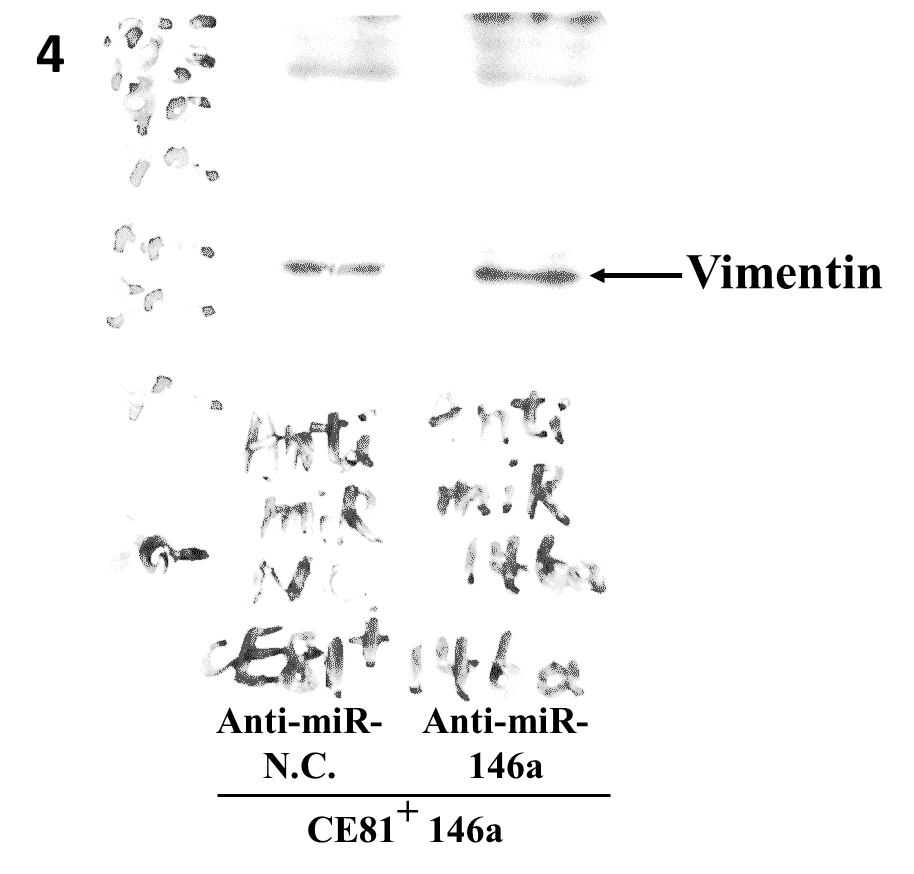
C**

**
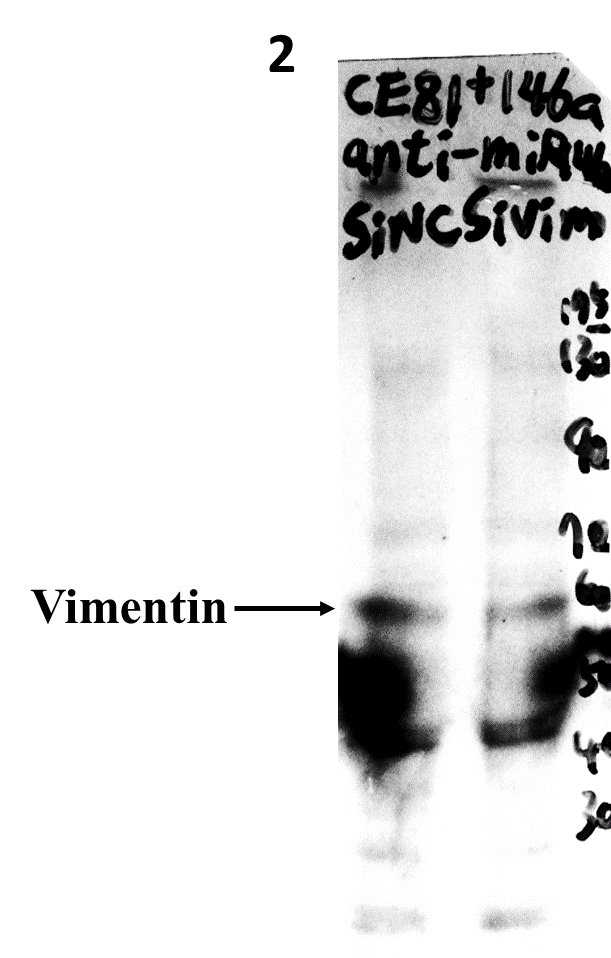
D**

**
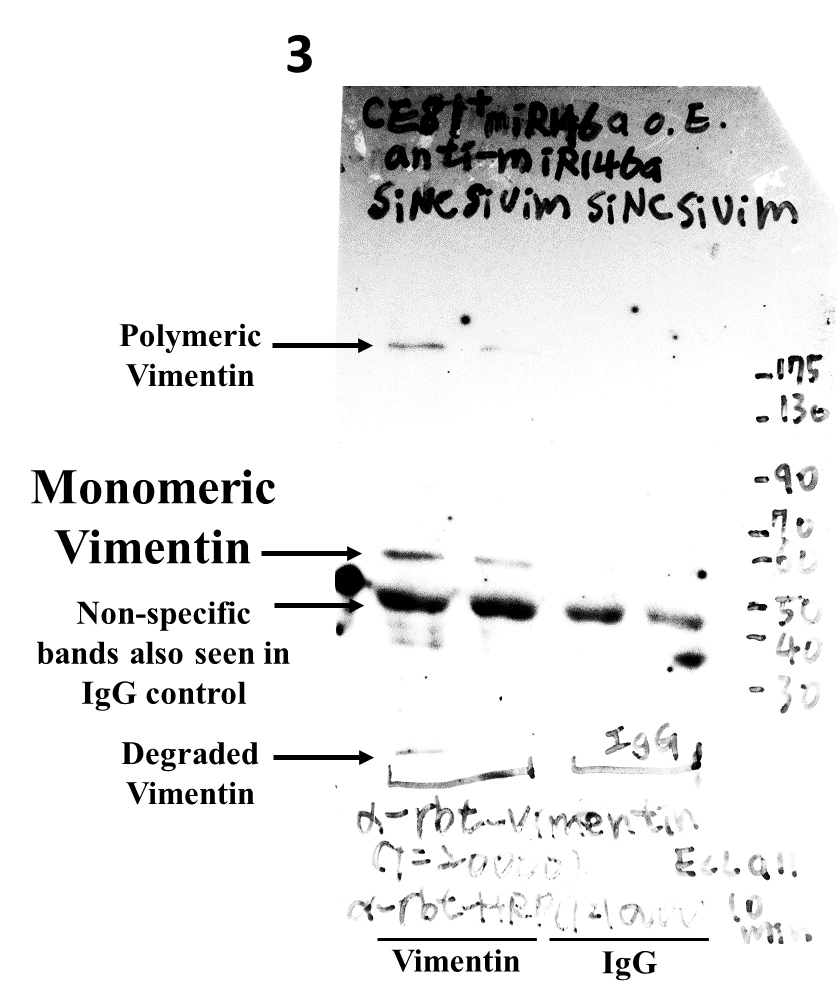

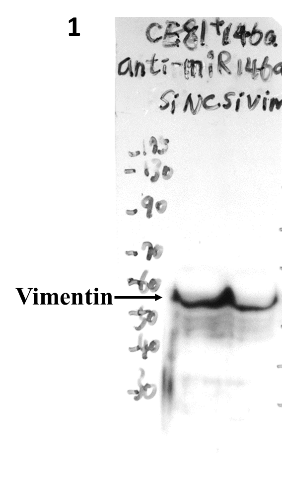
**

**Additional data legends**

**Additional Figure S1. Proliferation, migration and invasion of CE81^FN+^+CON and CE81^FN+^+146a cells.** (A) Cell proliferation of CE81^FN+^+CON and CE81^FN+^+146a was determined by MTT assay for 24, 48 and 96 hr. (B) The migration ability of CE81^FN+^+CON and CE81^FN+^+146a cells were investigated by Transwell™ migration assay at 48 hr. (C) The invasion ability of CE81^FN+^+CON and CE81^FN+^+146a cells were investigated by Transwell™ migration assay at 108 hr. The cell number was counted by Image J software. The data was analyzed by Student t test. ***: *p*<0.001.

**Additional Figure S2. *Mir-146a* targeting vimentin 3’-UTR was examined by luciferase reporter assay in HEK 293T cells.** The pre-miR-146a or its negative control (N.C.) was co-transfected with either wild-type or mutant-type of pMIR-Reporter-Vimentin 3’UTR plasmid into the HEK 293T cells. The luciferase activity was determined by a luminometer and normalized with Renilla luciferase activity (pRL-TK). The data were analyzed by Student’s t test. Statistically significant difference was indicated (***P<0.001; ns: no significance).

**Additional Figure S3. *MiR-146a* and negatively regulated vimentin affect ESCC KYSE cell migration.** (A) The expression levels of *miR-146a* in ESCC KYSE150 and 70 cells were measured by real-time PCR. (B) KYSE 150 cells were transiently transfected with 100 pmole/l of anti-*miR-146a* or scramble microRNA (anti-N.C.) using Lipofectamine™ for 48 hr followed by measuring the level of vimentin by Western blotting. (C) KYSE70 cells were transiently transfected with *miR-146a* or scramble microRNA (N.C.) followed by measuring the level of vimentin by Western blotting. β-actin was used as internal control. (D) The cells received the same treatment as (B and C) were used for Transwell™ migration assay. The quantitative data are shown by counting the number of the migrated cells on the bottom of the membrane after 48 hr. *P*-values were obtained by Student’s t test. **: *p<0.01*, ***: *p<0.001*. This experiment was repeated three times.

**Additional Figure S4. Protein expression of vimentin is negative regulation by *miR-146a.*** (A) Vimentin is downregulated by *miR-146a* relies on transient transfection of HEK293T cell and analyzes by Western blotting. Overexpression of vimentin in transiently transfected with pcDNA3.1-vimentin into HEK293T+146a stable cells compared to vector control. (B) The level of vimentin was increased in HEK293T+146a stable cells harboring with anti-*miR-146a* compared to the scramble anti-N.C. group. Up-regulation of vimentin in HEK293T+146a stable cells with anti-*miR-146a* can be suppressed by transiently transfected with si-vimentin.

**Additional Figure S5. *MiR-146a* level inversely correlates with ESCC patient tumor stages.** A total of 68 ESCC patient specimens were analyzed for *miR-146a* expression level by real-time PCR. The relative expression fold of *miR-146a* was normalized to U54. The relative expression levels of *miR-146a* was showed in figure which acculated form raw data of real-time PCR (Stage I and II *miR146a*/U54≧1.5 fold, and Stage III and IV < 1.5 fold). The stages of the ESCC patient specimens were determined by the pathologist in NCKU hospital. The data was analyzed by Student t test.

**Additional Figure S6**. **The hypothetic model of fibronectin assembly-mediated *miR-146a* suppressing ESCC cell mobility through targeting vimentin.** The fibronectin abundant assembling was negatively correlated with *miR-146a* expression. *MiR-146a* suppresses cell mobility by inhibition of vimentin.

**Additional Figure S7. Raw data of western blot images for Figure 4B and 4E in triplicate or quadruplicate.** Protein expression levels of vimentin in CE81^FN+^+CON and CE81^FN+^+146a stable cells were analyzed after transient transfection with various DNA constructs. (A) Quadruplicate results of vimentin expression levels in CE81^FN+^+CON stable cells after transient transfection with scrambled microRNA (miR-NC) or mimic-*miR-146a* as referred to the Figure 4B (left panel). (B) Quadruplicate results of vimentin expression levels in CE81^FN+^+CON cells after co-transfection with mimic-*miR-146a* and vector or pcDNA3-vimentin as referred to the Figure 4B (right panel). (C) Quadruplicate results of vimentin expression levels in CE81^FN+^+146a cells after transient transfection with scramble anti-N.C. or anti-*miR-146a* as referred to the Figure 4E (left panel). (D) Triplicate results of vimentin expression levels in the CE81^FN+^+146a stable cells after transient co-transfection with anti-*miR146a* and siN.C. or vimentin siRNA (si-vimentin) as referred to the Figure 4E (right panel).

**Additional Table S1 List of primers and sequences**

| **Usage** | **Primer name** | **Primer sequence** |
| --- | --- | --- |
| Real-time PCR | hsa-*miR-146a*  U54^b^  (For miRNA detection)  VIM  IGSF1  FBXL10  CASK  PBX2  UHRF1 | F: 5’-TGAGAACTGAATTCCATGGGTT-3’  F:5’-GGTACCTATTGTGTTGAGTAACGGTGA-3’  Paired with universal primer form Ncode^TM^ VILO^TM^ cDNA synthesis miRNA kit  F: 5’-CAGATGCGTGAAATGGAAGA-3’  R: 5’-TGGAAGAGGCAGAGAAATCC-3’  F: 5’-GCCTAATCGTGGTGGTTGTT-3’  R: 5’-GTGCCTGGTTCTCCTTCTTG-3’  F: 5’-TCAAACGCTGTGGAAACATC-3’  R: 5’-AAAGGAAAGTGTCGGCTCAC-3’  F: 5’-AGTTCTGGGGTGGTTTTGC-3’  R: 5’-ACCTTTCCAATCCCTTCAGC-3’  F: 5’-ATAGCCAAAGGGGTTCTGGT-3’  R: 5’-CACTGCCTCCACATCAAATG-3’  F: 5’-GGCTGTTGATGTTTCTGGTG-3’  R: 5’-GCCCGAGATGTTTCCTCTTT-3’ |
| ISH^a^ | hsa-*miR-146a* probe | 5’-AACCCATGGAATTCAGTTCTCA-3’ |
| Construct | 3’UTR of vimentin-wild type F  3’UTR of vimentin-wild type R  3’UTR of vimentin-mutant type F | 5’-ATAAGCTTAGTTAACAACCGACACTCC-3’  5’-ATACTAGTGAGTTTTTCCAAAGATTTATT  GAAG-3’  5’-ATAAGCTTACTACATTACAACCGACACTCC  TA-3’ |

F: forward primer; R: reverse primer; 3’UTR:3’-un-translated region

a: *In Situ* Hybridization

b: used as the internal control

**Additional Table S2. Correlation of *miR-146a* and vimentin protein expression with clinicopathologic parameters of sixty-eight ESCC patients**

| **Expression pattern** | **Age** | **Gender** | **Tumor stage**  **(I&II vs. III& IV)** | **Two-year survival** |
| --- | --- | --- | --- | --- |
| ***miR-146a*** | 0.9586 | 0.3666 | 0.1535 | P< 0.0001 |
| **vimentin** | 0.454 | 0.8665 | 0.3473 | P< 0.05 |
| ***miR-146a* & Vimentin** | 1 | 1 | 0.7133 | 0.4884 |

@ The association of age, gender, tumor stage, and two-year survival with level of *miR-146a*, vimentin or *miR-146a* & vimentin was analyzed by unpaired t test.

@ Multivariant analysis was conducted for the correlation of low-*miR146a* (*miR-146a* <0.8) plus high vimentin expression (Vimentin >1.8) with clinicopathologic parameters by Fisher's exact test.
